# Structural basis of selective cannabinoid CB_2_ receptor activation

**DOI:** 10.1038/s41467-023-37112-9

**Published:** 2023-03-15

**Authors:** Xiaoting Li, Hao Chang, Jara Bouma, Laura V. de Paus, Partha Mukhopadhyay, Janos Paloczi, Mohammed Mustafa, Cas van der Horst, Sanjay Sunil Kumar, Lijie Wu, Yanan Yu, Richard J. B. H. N. van den Berg, Antonius P. A. Janssen, Aron Lichtman, Zhi-Jie Liu, Pal Pacher, Mario van der Stelt, Laura H. Heitman, Tian Hua

**Affiliations:** 1 iHuman Institute, ShanghaiTech University, Shanghai, 201210 China; 2 School of Life Science and Technology, ShanghaiTech University, Shanghai, China; 3 Division of Drug Discovery and Safety, Leiden Academic Center for Drug Research, Leiden University, Oncode Institute, Leiden, the Netherlands; 4 Department of Molecular Physiology, Leiden Institute of Chemistry, Leiden University, Oncode Institute, Leiden, the Netherlands; 5 Laboratory of Cardiovascular Physiology and Tissue Injury, National Institute of Health/National Institute on Alcohol Abuse and Alcoholism, Rockville, MD USA; 6 Department of Pharmacology and Toxicology, Virginia Commonwealth University, Richmond, VA USA

**Keywords:** Electron microscopy, Small molecules, Receptor pharmacology

## Abstract

Cannabinoid CB_2_ receptor (CB_2_R) agonists are investigated as therapeutic agents in the clinic. However, their molecular mode-of-action is not fully understood. Here, we report the discovery of LEI-102, a CB_2_R agonist, used in conjunction with three other CBR ligands (APD371, HU308, and CP55,940) to investigate the selective CB_2_R activation by binding kinetics, site-directed mutagenesis, and cryo-EM studies. We identify key residues for CB_2_R activation. Highly lipophilic HU308 and the endocannabinoids, but not the more polar LEI-102, APD371, and CP55,940, reach the binding pocket through a membrane channel in TM1-TM7. Favorable physico-chemical properties of LEI-102 enable oral efficacy in a chemotherapy-induced nephropathy model. This study delineates the molecular mechanism of CB_2_R activation by selective agonists and highlights the role of lipophilicity in CB_2_R engagement. This may have implications for GPCR drug design and sheds light on their activation by endogenous ligands.

## Abstract (web summary)

Cannabinoid CB_2_ receptor (CB_2_R) agonists are investigated as therapeutic agents in the clinic. Here, authors report the discovery of LEI-102, a CB_2_R agonist, used in conjunction with three other CBR ligands (APD371, HU308, and CP55,940) to investigate selective CB_2_R activation.

## Introduction

**Fig. 1:**
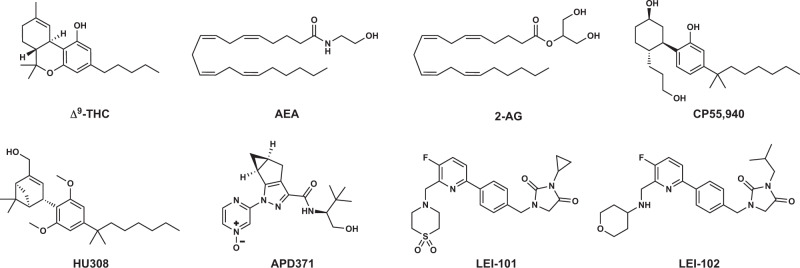
Chemical structures. Shown are the main constituent of Cannabis sativa Δ^9^-tetrahydrocannabinol (THC), and the two major endocannabinoids anandamide (AEA) and 2-arachidonoylglycerol (2-AG), as well as non-selective CBR agonist CP55,940 and CB_2_R agonists HU308, APD371, LEI-101, and LEI-102.

Preparations of the plant *Cannabis sativa* have been used for centuries in the treatment of various diseases, including cancer and neuropathic pain^1^. The synthetic version of its psychoactive constituent, Δ^9^-tetrahydrocannabinol (THC, Fig. 1), is in FDA approved drugs Marinol® or Syndros® (dronabinol). The extracted version of THC is one of the active constituents of oromucosal spray Sativex® (nabiximols). These drugs are primarily used for the treatment of chemotherapy-induced nausea, enhancement of appetite in cachexic AIDS-patients, and to alleviate the spasticity and pain associated with multiple sclerosis^2–6^. However, THC-based therapies are associated with clinically undesired psychotropic and cardiovascular adverse effects and challenging pharmacokinetic properties due to their high lipophilicity that may limit their therapeutic efficacy^7–10^.

THC exerts its therapeutic effects mostly via the G protein-coupled receptors (GPCRs) cannabinoid CB_1_ and CB_2_ receptors (CB_1_R and CB_2_R), which have 68% sequence identity in their seven transmembrane (TM) domains^11^. Both receptors are activated by the endogenous signaling lipids anandamide (AEA) and 2-arachidonoylglycerol (2-AG) (Fig. 1), the two main endocannabinoids. The CB_1_R, which is the most abundantly expressed GPCR in the central nervous system (CNS) is responsible for the psychotropic side effects of THC^12–14^. It plays a role in memory, learning, neurogenesis, neuronal migration, and synaptogenesis. Furthermore, its presence in many organ tissues belies more non-neurological functions^15^. The CB_2_R is mainly found on the cells of the immune system and is upregulated under pathophysiological conditions^16,17^. Its activation in general is associated with anti-inflammatory responses in tissue injury of the liver, heart, kidney, colon, and brain as determined in various preclinical models^18–22^. Based on preclinical studies, it is thought that selective CB_2_R agonists may retain and exceed certain therapeutic properties of THC without inducing psychotropic side effects^23^.

Various academic and industrial groups have developed selective CB_2_R ligands^24^. HU308 (Fig. 1) was the first selective CB_2_R agonist to be reported that displayed anti-inflammatory and analgesic properties in mouse models without inducing CNS-side effects^18^. However, poor physico-chemical properties (*e.g*. low solubility, high lipophilicity) of HU308, which has a calculated logarithm of octanol-water partition coefficient (cLogP) of 8.0^25^, and its analogs prevented the successful clinical translation of this class of cannabinoid-based drugs.

A next generation of CB_2_R ligands was developed with improved drug-like properties. For instance, Olorinab® (APD371, Fig. 1) is the most polar CB_2_R agonist reported to date with a cLogP of −0.4^26^. A phase 2a small-scale safety and tolerability trial in 14 patients with chronic abdominal pain associated with Crohn’s disease showed mild-to-moderate adverse events and an improvement in abdominal pain scores^27^. We have previously disclosed pyridinylbenzylimidazolidine-2,4-dione derivatives as selective CB_2_R agonists and studied their affinity, target binding kinetics and potency as a function of their lipophilicity, which resulted in the discovery of the orally available and peripherally restricted selective CB_2_R agonist LEI-101 (Fig. 1)^28–30^. It is intriguing that the CB_2_R binding pocket tolerates a wide array of ligands with very different scaffolds and hydrophobicity. For example, HU308 has a 2-billion-fold higher lipophilicity than APD371. Despite the tremendous progress in the field of CB_2_R drug discovery, we still do not have any molecular understanding on how these CB_2_R agonists selectively activate CB_2_R over CB_1_R.

Recently, three-dimensional structures of the CB_1_R and CB_2_R have been elucidated in both the active and inactive states by crystallography or cryo-electron microscopy (cryo-EM) and the binding modes of diverse ligands and their activation mechanism were reported^31–35^. Remarkably, those structures revealed that CB_1_R and CB_2_R possess a highly similar, lipophilic orthosteric agonist binding pocket, which makes it challenging to explain the selective activation of CB_2_R. To date, no structural studies with selective CB_2_R agonists have been reported that could aid in understanding the molecular basis of CB_2_R selectivity.

Here, we present the discovery of LEI-102, a potent and selective CB_2_R agonist with good physico-chemical and biological properties. LEI-102 is used in conjunction with CB_2_R selective agonists APD371 and HU308, and non-selective agonist CP55,940 to investigate the activation mechanism of CB_2_R. For this study, we combine ligand-target binding kinetics, site-directed mutagenesis, and cryo-EM methods. We find that CB_2_R has a distinct activation mechanism compared to CB_1_R. Additionally, we find that the physico-chemical properties of the ligands influence their entry pathway into the receptor. Highly lipophilic ligands, such as HU308 and the endocannabinoids, may reach the binding pocket through the membrane, whereas more polar ligands, such as LEI-102, APD371 and CP55,940, enter the receptor via an alternative route. Furthermore, we show that the favorable physico-chemical properties of LEI-102 and CB_2_R selectivity underscore its promising in vivo efficacy via oral administration in a chemotherapy-induced nephropathy model without inducing CNS-mediated side effects. Together, these studies enhance our insights into how certain physico-chemical properties of ligands translate to in vivo activity and changes their engagement to GPCRs.

## Results

### LEI-102 as a high affinity and potent CB_2_R-selective agonist

To obtain a selective CB_2_R agonist with beneficial physico-chemical properties, LEI-102, a pyridinylbenzylimidazolidine-2,4-dione derivative, was designed and synthesized (Supplementary Fig. 1). LEI-102 combined an isobutyl substituent on the imidazolidine with an aminotetrahydropyran to replace the cyclopropyl and thiomorpholine 1,1-dioxide in LEI-101, respectively^30^. LEI-102 had a cLogP of 2.1 as calculated by ChemDraw 19.0 (Supplementary Table 1). The inhibitory constant (pK_i_), potency (pEC_50_) and intrinsic activity (E_max_) of LEI-102 were determined in [^3^H]RO6957022 displacement assays on stably expressing CB_2_R membranes and [^35^S]GTPγS G protein activation assays using HEK293T membranes transiently expressing recombinant hCB_2_R or hCB_1_R, respectively (Supplementary Table 2). APD371, HU308, CP55,940 and the endocannabinoids AEA and 2-AG were also explored. LEI-102 had a high binding affinity for CB_2_R (pK_i_ = 8.0 ± 0.1) and was more potent than the selective CB_2_R agonists APD371 and HU308. LEI-102 did not bind CB_1_R, thereby showing at least 1000-fold selectivity (Supplementary Table 3). In G protein activation assays, LEI-102 activated the receptor as a partial agonist (E_max_ 76 ± 1%) with a pEC_50_ value of 6.9 ± 0.2 (Supplementary Table 2).

### Distinct target binding kinetic profiles of CB_2_R agonists

To quantify the ligand-target binding kinetic parameters of the agonists in more detail, we performed displacement and competition association assays with [^3^H]RO6957022 on membranes stably expressing hCB_2_R (Supplementary Table 2). The equilibrium K_i_ and kinetic K_D_ values were well correlated, validating the competition association assay. First, we determined the dissociation rate constants (*k*_off_) of all agonists and converted these into a residence time (RT). LEI-102 had a RT of 16 min, which was around half that of APD371 (45 min) and CP55,940 (32 min), whereas HU308 had the longest RT at the receptor of 71 min (Supplementary Table 2). Endocannabinoids 2-AG and AEA had the shortest RT, both approximately 7 min. Of note, we found that the association rate constants (*k*_on_) varied greatly between the different agonists, ranking from fast to slow engagement CP55,940 > LEI-102 > 2-AG > APD371 > HU308 = AEA. The calculated engagement time (ET) to CB_2_R at 1 µM of each agonist further emphasized that CP55,940 arrived at CB_2_R within one second, whereas APD371, LEI-102, and 2-AG needed between 16 and 40 s to reach the CB_2_R binding site. Interestingly, HU308 and AEA took 143 and 152 s to bind CB_2_R, respectively. In view of the distinct target-binding kinetic profiles of the four synthetic CB_2_R agonists, we decided to elucidate their binding poses in CB_2_R using cryo-EM method.

### Overall similar structural comparison of CB_2_R-G_i_ in complex with different agonists

**Fig. 2:**
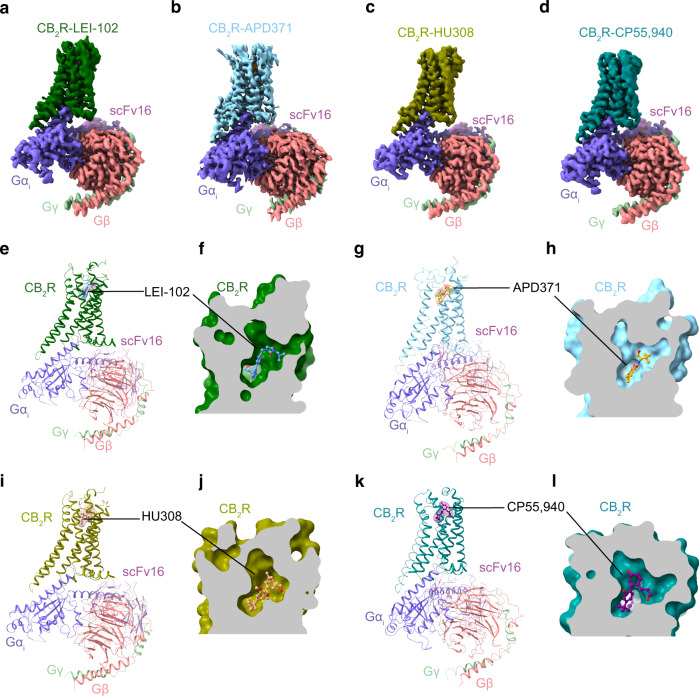
Cryo-EM structures of CB_2_R-G_i_ complexes. Cryo-EM density maps of **a** LEI-102 (Dark green), **b** APD371 (Sky blue), **c** HU308 (Olive), and **d** CP55,940 (Teal) bound CB_2_R in complex with Gα_i_ (Slate), Gβ (Salmon), Gγ (Pale green), scFv16 (Violet purple). **e**–**l** Overall structures of CB_2_R-G_i_ complexes and enlarged view of orthosteric pocket of **f** LEI-102, **h** APD371, **j** HU308, and **l** CP55,940 using the same color codes as (**a**–**d**), with agonists shown as cornflower blue (LEI-102), orange (APD371), dark salmon (HU308) and purple (CP55,940) sticks, respectively.

To obtain the stable complex sample of CB_2_R-G_i_ bound with LEI-102, APD371, HU308, or CP55,940, a similar procedure was used as for our previous AM12033-CB_2_R-G_i_ complex preparation (PDB: 6KPF). Single particle analysis of the cryo-EM samples yielded a normal global map for CB_2_R-LEI-102-G_i_-scFv16, CB_2_R-APD371-G_i_-scFv16, CB_2_R-HU308-G_i_-scFv16, and CB_2_R-CP55,940-G_i_-scFv16, complex, at 2.9, 3.0, 3.0, and 2.9 Å, respectively (Fig. 2, Supplementary Table 4, and Supplementary Figs. 2–5). The ligand, receptor and G protein in the isolated complex were clearly visible in the cryo-EM maps (Fig. 2 and Supplementary Fig. 6). The overall structures of the four complexes were comparable, with root mean square deviation (RMSD) of the Cα atoms of the receptors around 0.35 Å. The ligand binding interfaces of the four CB_2_R and G_i_ complexes were similar to each other, and to those of the previous AM12033-CB_2_R-G_i_ or WIN55212-2-CB_2_R-G_i_ complex structures.

### The binding mode of LEI-102 in CB_2_R

**Fig. 3:**
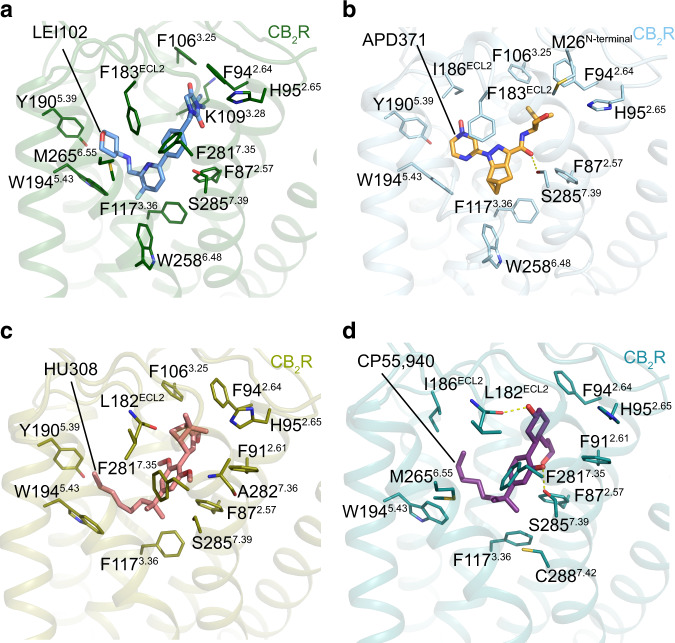
Key interactions between CB_2_R and agonists. Key residues involved in **a** LEI-102, **b** APD371, **c** HU308, and **d** CP55,940 binding in CB_2_R-G_i_ complex structures. The amino acids involved in interactions are shown as sticks, hydrogen bonds are highlighted with yellow dashed lines. Same color codes as in Fig. 2.

A clear electron density in the orthosteric ligand binding pocket in the LEI-102-CB_2_R-G_i_ complex resulted in the unambiguously defined binding pose of LEI-102 (Supplementary Fig. 6a). LEI-102 predominantly interacted with the residues in the binding pocket via hydrophobic interactions (Fig. 3a and Supplementary Fig. 7a). The isobutyl substituent of LEI-102 showed interactions with residues S90^2.60^ (Ballesteros-Weinstein numbering in superscript), F106^3.25^, K109^3.28^, and I110^3.29^ in CB_2_R. The imidazolidine-2,4-dione formed a π-π interaction with F94^2.64^ and showed further hydrophobic interactions with F106^3.25^ and P184^ECL2^. The benzyl formed an aromatic interaction with F183^ECL2^, and hydrophobic interactions with F87^2.57^ and S285^7.39^. The phenyl ring in the core of LEI-102 formed a cation–π interaction with F183^ECL2^ and T-shaped π-π interaction with F281^7.35^. The pyridine had hydrophobic contacts with F117^3.36^ and W258^6.48^. The aminotetrahydropyran sidechain protruded into the long channel and formed hydrophobic interactions with residues I110^3.29^, T114^3.33^, I186^ECL2^, Y190^5.39^, L191^5.40^, W194^5.43^, and M265^6.55^. Additionally, a hydrogen bond was formed with T114^3.33^ (Supplementary Fig. 7a).

### The binding mode of APD371 in CB_2_R

APD371 mainly formed hydrophobic and aromatic interactions with residues from ECL2, TM2, TM3, TM5, TM6, and TM7 (Fig. 3b and Supplementary Fig. 7b). The carbonyl group of APD371 formed a putative hydrogen bond with S285^7.39^ and a hydrophobic interaction with F87^2.57^. The pyrazole and pyrazine cores of APD371 formed aromatic interactions with F183^ECL2^. Furthermore, the pyrazine core formed hydrophobic contacts with T114^3.33^, I186^ECL2^, L191^5.40^, and W194^5.43^. The (*S*)-1-hydroxy-3,3-dimethylbutyl head formed hydrophobic contacts with residues M26^N-terminus^, S90^2.60^, F94^2.64^, F106^3.25^, I110^3.29^ and V113^3.32^. The cyclopropyl group formed hydrophobic contacts with F117^3.36^, W194^5.43^, W258^6.48^, and V261^6.51^.

### The binding mode of HU308 in CB_2_R

The interactions between HU308 and CB_2_R were hydrophobic, including residues from ECL2, TM2, TM3, TM5, TM6, and TM7 (Fig. 3c and Supplementary Fig. 7c). The phenyl of 2,6-dimethoxyphenyl core formed hydrophobic interactions with F87^2.57^, F183^ECL2^, and S285^7.39^, the C2-methoxy formed hydrophobic contacts with A282^7.36^ and S285^7.39^, and the C6-methoxy formed hydrophobic contacts with I110^3.29^, V113^3.32^, and T114^3.33^, respectively. The dimethylheptyl chain of HU308 extended into the long channel and formed hydrophobic interactions with residues from ECL2 (F183^ECL2^), TM3 (T114^3.33^, F117^3.36^) and TM5 (W194^5.43^). The 1,1-dimethyl formed hydrophobic interactions with residues F87^2.57^, F117^3.36^, F281^7.35^, and S285^7.39^. The bicyclic head of HU308 formed hydrophobic interactions with M26^N-terminus^, F106^3.25^, I110^3.29^, S90^2.60^, F94^2.64^, P184^ECL2^, and the 2-methanol formed a hydrophobic interaction with F94^2.64^ (Supplementary Fig. 7c).

### The binding mode of CP55,940 in CB_2_R

CP55,940 adopted an L-shape conformation in the orthosteric binding pocket (Fig. 3d and Supplementary Fig. 6d). The cyclohexanol group formed hydrophobic interactions with F94^2.64^, L182^ECL2^, F183^ECL2^, and P184^ECL2^. The hydroxyl group established a hydrogen bond with L182^ECL2^ and the hydroxypropyl formed hydrophobic contacts with F87^2.57^, S90^2.60^, F91^2.61^, I110^3.29^, and V113^3.32^. The phenol core formed hydrophobic interactions with F87^2.57^, F183^ECL2^, F281^7.35^, and S285^7.39^, and its hydroxyl additionally formed a hydrogen bond with S285^7.39^. The dimethyl formed hydrophobic interactions with F183^ECL2^, F281^7.35^, M265^6.55^, F87^2.57^, F117^3.36^, and C288^7.42^. The dimethylheptyl alkyl chain of CP55,940 extended into the long channel and formed hydrophobic interactions with residues I110^3.29^, F183^ECL2^, I186^ECL2^, W194^5.43^, T114^3.33^ and F117^3.36^ (Supplementary Fig. 7d).

### LEI-102 and APD371 require H95^2.65^ for G protein activation in CB_2_R

**Fig. 4:**
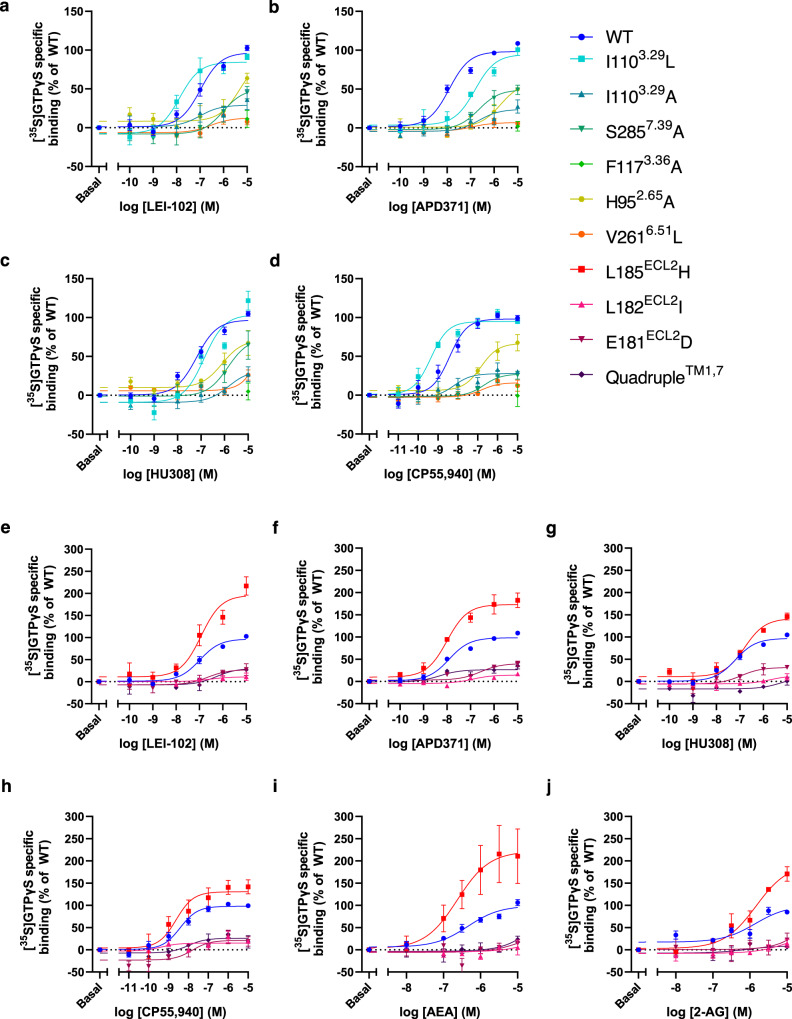
Characterization of G protein activation of wild type (WT) and mutant CB_2_R by synthetic agonists and endocannabinoids. Dose-response curves for G protein activation of WT and mutants that are located in the CB_2_R binding pocket by **a** LEI-102, **b** APD371, **c** HU308, and **d** CP55,940. **e**–**j** Dose response curves for G protein activation of WT and mutants that are proposed to be involved in ligand entry of CB_2_R via either the ECL2 or membrane access by **e** LEI-102, **f** APD371, **g** HU308, **h** CP55,940, **i** AEA and **j** 2-AG. **a**–**j** The maximum activation level of WT CB_2_R was set to 100% while the basal levels were set to 0%. Data are presented as mean ± SEM of at least three individual experiments performed in duplicate (specific *n* values are given in Supplementary Table 6). Source data are provided as a Source Data file.

To study the mechanism of CB_2_R activation, five residues in the binding pocket were further characterized based on the complex structures (Fig. 3). Six CB_2_R mutants were created, i.e. four residues (S285^7.39^, H95^2.65^, I110^3.29^, and F117^3.36^) were replaced by alanine, as these are conserved between CB_2_R and CB_1_R, and two others (I110^3.29^, V261^6.51^) were substituted by the hCB_1_R reciprocal residue leucine. All mutants were sufficiently expressed at the cell surface as determined with an ELISA (Supplementary Fig. 8, Supplementary Table 5). To characterize the binding mechanisms of LEI-102, APD371, HU308, and CP55,940, their responses were investigated by [^3^H]CP55,940 displacement and [^35^S]GTPγS binding assays. Of note, in the [^3^H]CP55,940 displacement assay, only the CB_2_R-I110^3.29^L mutant showed a sufficient binding window (data not shown). This prevented the affinity determination of the four agonists on other mutant receptors. Five mutant receptors, except CB_2_R-F117^3.36^A, were still active in the [^35^S]GTPγS functional assay, thereby allowing us to study the receptor activation mechanism (Fig. 4a–d and Supplementary Table 6). All four synthetic agonists were unable to activate CB_2_R-F117^3.36^A, which indicated an important role of this residue in the activation of CB_2_R.

The potency of LEI-102 was significantly increased at the CB_2_R-I110^3.29^L mutant to a pEC_50_ value of 7.8 ± 0.1 in the G protein activation assay, while the binding affinity remained similar to wild type (WT) receptor (Fig. 4a and Supplementary Tables 6 and 7). Three mutations CB_2_R-I110^3.29^A, CB_2_R-S285^7.39^A and CB_2_R-V261^6.51^L had no significant effect on the potency of LEI-102 in the functional assay. In contrast, the potency on mutant receptor CB_2_R-H95^2.65^A was significantly reduced for LEI-102. No gain in binding affinity for the swap mutant in CB_1_R-L359^6.51^V was found with LEI-102 (Supplementary Tables 3 and 8).

APD371 acted as a full CB_2_R agonist with a pEC_50_ value of 7.9 ± 0.1 and a higher maximal activation compared to that of CP55,940 in the functional assay (Supplementary Table 2). Mutant receptor CB_2_R-I110^3.29^L did not affect the G protein response of APD371 (Fig. 4b and Supplementary Table 6), while the binding affinity was significantly reduced to a pK_i_ of 7.1 ± 0.0 (Supplementary Table 7). APD371 potency was not affected by mutant receptors CB_2_R-I110^3.29^A or CB_2_R-S285^7.39^A. The responses of APD371 for CB_2_R-H95^2.65^A and CB_2_R-V261^6.51^L were significantly impacted with 158-fold and 10-fold drop in potency, respectively (Supplementary Table 6).

Thus, we uncovered a crucial role for CB_2_R-H95^2.65^ in G protein activation of CB_2_R by LEI-102 and APD371. Furthermore, LEI-102 activation was increased for the CB_2_R-I110^3.29^L mutant, while APD371 activation relied on CB_2_R-V261^6.51^.

### An important role for S285^7.39^ and V261^6.51^ in CB_2_R activation by HU308 and CP55,940

The potency and affinity of HU308 on CB_2_R were not affected by the CB_2_R-I110^3.29^L swap mutant (Fig. 4c and Supplementary Tables 6, 7). In addition, activation of mutant receptors CB_2_R-I110^3.29^A and CB_2_R-H95^2.65^A by HU308 was not affected with pEC_50_ values of 6.4 ± 0.5 and 6.6 ± 0.6, respectively. The maximum activation level of mutant receptor CB_2_R-S285^7.39^A was unaffected compared to WT receptor, but a significant 15-fold loss in potency was observed. Lastly, CB_2_R-V261^6.51^L had a significant loss of potency, i.e. more than 120-fold lower (Fig. 4c and Supplementary Table 6).

Similar to HU308, the potency of CP55,940 on CB_2_R was not affected by the CB_2_R-I110^3.29^ mutations compared to WT in the G protein activation assay, nor was its binding affinity for CB_2_R-I110^3.29^L (Fig. 4d and Supplementary Tables 6, 7). In response to CP55,940, mutant receptors CB_2_R-S285^7.39^A and CB_2_R-V261^6.51^L were significantly affected with decreased pEC_50_ values of 6.7 ± 0.1 and <5, respectively. Moreover, the potency of CP55,940 was significantly affected on the CB_2_R-H95^2.65^A with a 40-fold decrease compared to WT receptor (Fig. 4d and Supplementary Table 6). No gain in potency or affinity was observed for the swap mutant CB_1_R-L359^6.51^V for either HU308 or CP55,940 (Supplementary Tables 3 and 8).

Taken together, this showed that CB_2_R-S285^7.39^ and CB_2_R-V261^6.51^ were crucial for HU308 and CP55,940 to activate the G protein at CB_2_R, where CP55,940 additionally required an interaction with CB_2_R-H95^2.65^.

### HU308 and endocannabinoids gain access via membrane entry

Our detailed ligand-target binding kinetic analysis revealed that the highly lipophilic HU308 and anandamide had a very slow on-rate compared to the other ligands. Since it has previously been postulated that ligands of lipid receptors may gain access to the binding pocket via a membrane channel, we examined two potential ligand entry pathways at CB_2_R, i.e. either via ECL2 or via a membrane channel in TM1 and TM7. To this end, four additional mutant receptors were created. Three residues in the ECL2 of CB_2_R, which were different from CB_1_R, were mutated towards the reciprocal CB_1_R residues, i.e. CB_2_R-L185^ECL2^H, CB_2_R-L182^ECL2^I, and CB_2_R-E181^ECL2^D. In the fourth mutant receptor, four residues in TM1 and TM7 that align the potential membrane channel in CB_2_R were mutated to the reciprocal CB_1_R residues and combined as a quadruple mutant, i.e. CB_2_R-K279^7.33^T, CB_2_R-K33^1.32^Q, CB_2_R-V36^1.35^I and CB_2_R-C40^1.39^S (termed “CB_2_R-Quadruple^TM1,7^”). Next, we tested all four synthetic agonists and the two endocannabinoids on these four CB_2_R mutant receptors in [^3^H]CP55,940 and [^35^S]GTPγS assays. Only CB_2_R-L185^ECL2^H and CB_2_R-Quadruple^TM1,7^ were evaluated in the [^3^H]CP55,940 displacement assays due to insufficient binding window for the other two mutant receptors (data not shown). The binding affinities of the agonists were not affected for mutant receptors CB_2_R-L185^ECL2^H and CB_2_R-Quadruple^TM1,7^ (Supplementary Table 7). Interestingly, the potencies of LEI-102, APD371, and CP55,940 in the functional assay were not significantly affected for any of the mutant receptors, whereas HU308 and the endocannabinoids were less potent on CB_2_R-L182^ECL2^I (Fig. 4e–j and Supplementary Table 6). Additionally, the endocannabinoids showed a decreased potency on CB_2_R-L181^ECL2^D, but not on CB_2_R-L185^ECL2^H. Of note, HU308 and both endocannabinoids completely lost their ability to activate CB_2_R in the CB_2_R-Quadruple^TM1,7^ mutant, suggesting that this may be an important access point to the receptor binding pocket for these agonists (Fig. 4g, i, j).

### LEI-102 attenuates cisplatin-induced nephrotoxicity without CB_1_R-mediated side effects

**Fig. 5:**
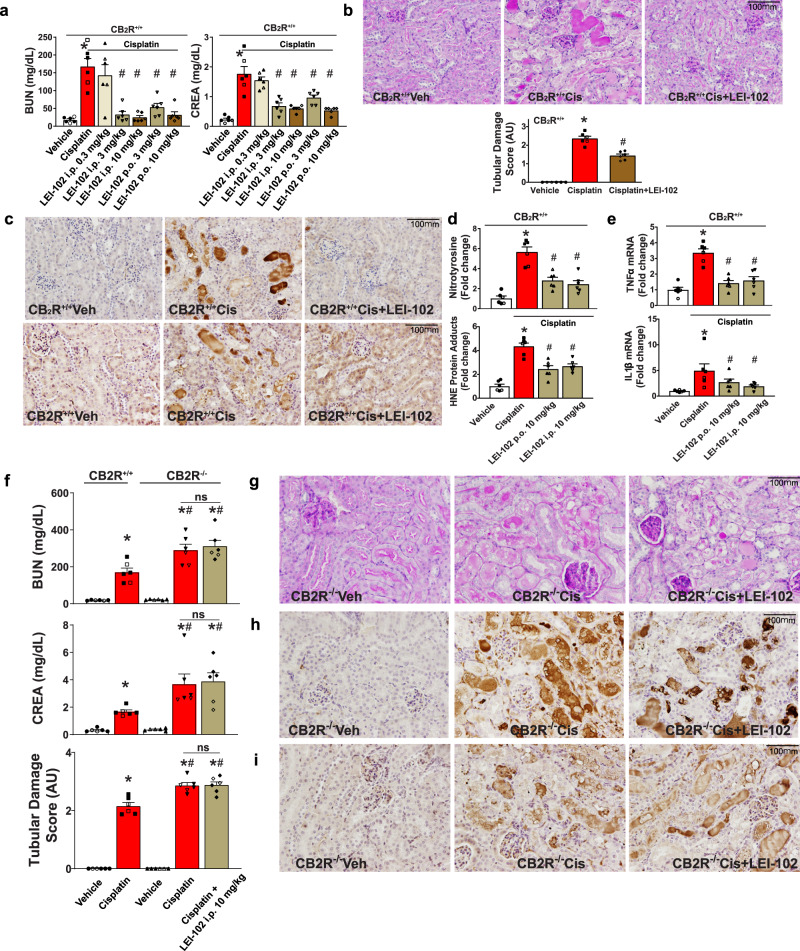
CB_2_R agonist LEI-102 attenuates cisplatin-induced renal dysfunction, oxidative stress, and inflammation in a CB_2_R-dependent manner. **a** Cisplatin-induced renal dysfunction 72 h after administration to mice as evidenced by increased serum levels of blood urea nitrogen (BUN) and creatinine (CREA), which were attenuated by CB_2_R agonist LEI-102 in a dose-dependent manner when administered either i.p. or p.o. (*p < 0.001 vs. vehicle group, #*p* < 0.001 vs. cisplatin group). **b** Periodic Acid-Schiff (PAS) staining in representative kidney sections from cisplatin treatment samples showing protein cast, vacuolation, and desquamation of epithelial cells in the renal tubules which are attenuated with LEI-102. Tubular damage score from kidney sections is shown (*p < 0.001 vs. vehicle group, #*p* < 0.001 vs. cisplatin group). **c** The cisplatin-induced nitrative and oxidative stress (nitrotyrosine staining (top row) and HNE staining (bottom row)) in representative kidney sections were also attenuated by LEI-102. This was confirmed by quantitative determination of protein nitration and HNE adducts formation by ELISA (**d**) (*p < 0.001 vs. vehicle group, #*p* < 0.001 vs. cisplatin group). **e** The cisplatin-induced kidney pro-inflammatory cytokine expressions were also attenuated by the CB_2_R agonist. (*p < 0.001 vs. vehicle group, #*p* < 0.05 vs. cisplatin group). The protective effects of LEI-102 on cisplatin-induced kidney dysfunction (BUN and CREA) and tubular injury (tubular damage score) (**f**) (*p < 0.001 vs. vehicle WT or KO group, *#p* < 0.05 vs. cisplatin WT group), histopathological injury (**g**), nitrative (**h**) and oxidative stress (**i**) were abolished in CB_2_R knockout mice. All results are means ± SEM of *n* = 6/group for panels **a**, **b**, **d**, **e**, **f** Closed and open symbols are used for male and female mice respectively (4 males and 2 females/group). In panels **a**, **b**, **d**, and **e** one-way ANOVA followed by Tukey’s post hoc test for multiple comparisons were used, in panel **f** unpaired two-tailed t-test was used. The analysis was conducted using GraphPad Prism 6 software. *p* < 0.05 was considered statistically significant (the exact *p* values are indicated in the supplemental data).

In view of the excellent physico-chemical properties of LEI-102 and its selective CB_2_R agonist profile, we investigated the compound in a well-established in vivo model of kidney inflammation and injury induced by cisplatin. In this model, CB_2_R activation is associated with protective effects^29^. Cisplatin (25 mg/kg, i.p.) induced marked elevations of serum creatinine and blood urea nitrogen levels (functional markers of kidney injury) 72 h following cisplatin injection in wild type mice compared with vehicle-treated control animals. LEI-102 showed a dose-dependent attenuation of the functional markers of cisplatin-induced kidney injury both when administered p.o. (orally) or i.p. (Fig. 5a). Renal dysfunction was also accompanied by morphological damage to the kidney tubules determined by histological examination following PAS staining. LEI-102 (10 mg/kg) significantly decreased tubular injury as determined by this staining (Fig. 5b). Marked increases in oxidative and nitrative stress markers (4-HNE and 3-nitrotyrosine) were observed in kidneys of cisplatin-treated mice determined by immunostaining and quantitative ELISA. Furthermore, LEI-102 (10 mg/kg by i.p. or p.o.) decreased lipid peroxidation and protein nitration (Fig. 5c, d). Additionally, the pro-inflammatory cytokines TNFα and IL1β that were elevated due to the cisplatin-induced injury were attenuated in LEI-102 treated mice (Fig. 5e). Importantly, the protective effects of LEI-102 against cisplatin-induced renal dysfunction and tubular damage (Fig. 5f), histopathological injury (Fig. 5g) and markers of oxidative-nitrative stress (Fig. 5h, i) were abolished in CB_2_R knockout mice, which had enhanced kidney injury/dysfunction compared to their wild types.

**Fig. 6:**
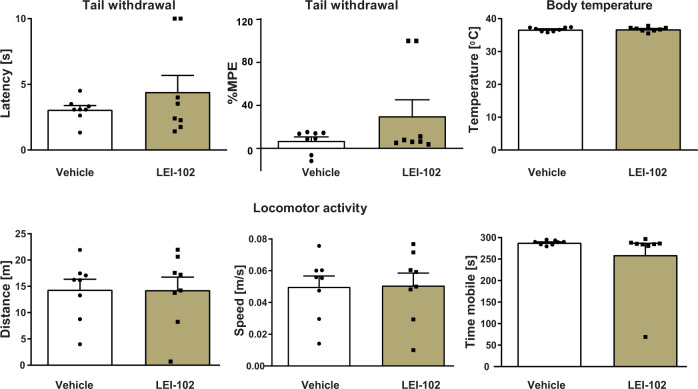
The CB_2_R agonist LEI‐102 does not induce cannabimimetic CB_1_R-mediated effects (Tetrad assay) in vivo. LEI‐102 (25 mg/kg, p.o.) did not affect nociceptive behavior assessed in tail withdrawal test and body temperature, as compared with mice receiving vehicle (upper row). No effects on locomotor behavior were found (lower row). Results are means ± SEM; *n* = 8 per group.

To determine whether LEI-102 maintained its selectivity for CB_2_R over CB_1_R in vivo, LEI-102 was tested in the mouse tetrad assay for CB_1_R activity^18^. In this assay, four consecutive behavioral tests, related to anti-nociception, hypothermia, catalepsy, and spontaneous activity, were performed 120 min after administration of the agonist. LEI-102 (25 mg/kg, p.o.) did not produce any effects in the tetrad assay as compared with vehicle. There were no effects on nociceptive behavior assessed in tail withdrawal test nor on body temperature (Fig. 6 upper row). No effect was found on locomotor behavior (Fig. 6 lower row) in case of distance traveled, time spent mobile, or running speed of mice. Nor was catalepsy observed following administration of LEI-102. These results indicated that LEI-102 (or one of its metabolites) did not produce CB_1_R-mediated CNS-side effects at doses up to 25 mg/kg (p.o.). Hence, the CB_2_R agonist LEI-102 maintained its selectivity over CB_1_R in vivo.

## Discussion

So far, several crystal and cryo-EM CBR structures have been resolved in which non-selective agonists adopt a nearly identical binding position in the orthosteric pocket, regardless of the receptor^34–38^. In this study, we aimed to generate a better understanding of the binding and activation mechanism of CB_2_R-selective agonists. Therefore, we combined ligand-target binding kinetics, site-directed mutagenesis, and cryo-EM studies to investigate the activation mechanism of CB_2_R for the introduced CB_2_R selective agonist LEI-102 supplemented with agonists APD371, HU308, and CP55,940 on a molecular level. Furthermore, we investigated potential hotspots for CB_2_R/CB_1_R selectivity by creating swap mutants and discovered a ligand entry pathway for CBR agonists and endocannabinoids.

First, our data revealed a crucial role for CB_2_R-F117^3.36^ as replacement by alanine resulted in a complete loss of G protein activation by all tested agonists (Fig. 4a–d and Supplementary Table 6). It has been shown that the CB_1_R counterpart F200^3.36^ plays an important regulatory role in activation as part of the “twin toggle switch” with CB_1_R-W356^6.48^^39^. In contrast, CB_2_R-W258^6.48^ has been described to be solely responsible for activation as a toggle switch without the help of CB_2_R-F117^3.36^ in structural studies, since the conformation of CB_2_R-F117^3.36^ in agonist-bound structures is comparable to the conformation in the antagonist-bound CB_2_R structure as well as the CB_1_R agonist-bound structures^33,36^. Our mutation data further supports this hypothesis, as we do not see the same constitutive activity pattern (Supplementary Table 6) as observed by McAllister et al. for the reciprocal CB_1_R-F220^3.36^ excluding CB_2_R-F117^3.36^ from a suppressive function^39^. Together, this data provides evidence for a different, but important, role for F117^3.36^ in CB_2_R activation.

In CB_1_R, water-mediated interactions between CB_1_R-H178^2.65^, CB_1_R-S383^7.39^, and bound ligands have previously been shown with in silico modeling^40,41^. The importance of CB_1_R-S383^7.39^ for classical synthetic cannabinoids such as AM11542, AM841, and CP55,940 was further emphasized in CB_1_R-S383^7.39^A mutants^36^. This is in line with the observation that removal or methylation of the phenolic OH on classical cannabinoids, such as in L-759656, JWH-133, and HU308, always affords selectivity over CB_1_R^18,42^. Non-classical agonists, such as WIN55,212-2, do not form a hydrogen bond with CB_1_R-S383^7.39^ and consequently are not affected by an alanine mutation^43^. This translates to our results that CP55,940 and HU308 are more affected by the CB_2_R-S285^7.39^A mutation than LEI-102 and APD371 (Fig. 4a–d and Supplementary Table 3). The decrease in activation is at least 30-fold smaller for CB_2_R than CB_1_R^36^. The elucidated cryo-EM structures of our four agonists did not show direct interactions with CB_2_R-H95^2.65^, though we cannot rule out its role in stabilizing the surrounding residues. The large effect seen on G protein activation of CB_2_R-H95^2.65^A by LEI-102, APD371, and CP55,940 (Fig. 4a–d and Supplementary Table 6) must therefore stem from an indirect interaction, supporting the polar network hypothesis between CB_2_R-H95^2.65^ and CB_2_R-S285^7.39^ in CB_2_R.

Residues at position 6.51 have previously been described to be involved in the binding sites of µ, δ, and κ opioid receptors, the dopamine D2 receptor, and adenosine receptors, and could play a role in ligand binding selectivity between different subtypes^44–46^. In our studies, introduction of the bulkier CB_1_R leucine on this position in CB_2_R-V261^6.51^L reduced the G protein activation by APD371, HU308, and CP55,940, while LEI-102 could still be accommodated in the binding pocket (Fig. 4a–d and Supplementary Table 6). Furthermore, with the swap mutant CB_1_R-L359^5.61^V we found a trend in partial recovery of displacement of [^3^H]CP55,940 by the CB_2_R selective agonists LEI-102, HU308, and APD371, although not significant (Supplementary Table 3). This supports a role of this residue in the selectivity of agonists in CB_2_R.

The ECL2 has frequently been implicated to be important for GPCR activation and some GPCRs even use their ECL2 as a ligand to auto-activation^47^. There are distinct differences between the conformations of ECL2 in CB_1_R and CB_2_R. In antagonist-bound CB_1_R crystal structures, the ECL2 dips into the binding pocket, interacting with the ligand and inducing the inactive conformation^31,32^. The inactive state of CB_2_R, however, does not expand like CB_1_R and instead the ECL2 acts more as a lid on the binding pocket in active and inactive CB_2_R, akin to active CB_1_R^33^. A key distinction seen in the CB_1_R crystal structures with AM6538 and taranabant, is the ionic lock formed by CB_1_R-E100^N-terminus^ (CB_2_R-L17) and CB_1_R‑H270^ECL2^ (CB_2_R-L185)^31,32^. We observed improved binding of [^3^H]CP55,940 for LEI-102 and HU308 with the CB_1_R-H270^ECL2^L mutation, while the non-selective agonists showed no change (Supplementary Table 3). Through the loss of this ionic lock, selectivity over CB_1_R is partially lost, showing that the expulsion of ECL2 upon ligand entry may play an important role in selectivity.

In recent years, computational studies have suggested that lipophilic ligands for various GPCRs, such as the opsin receptor, sphingosine-1-phosphate receptor 1 (S1P_1_) and cannabinoid receptors, might gain access to the binding pocket through lateral diffusion via a membrane channel between TM1 and TM7^32,41,48–51^. We experimentally examined this membrane entry pathway by creating a CB_2_R quadruple mutant (K33^1.32^Q, V36^1.35^I, C40^1.39^S, and K279^7.33^T) for which we observed a significant loss of potency and a corresponding trend in reduced affinity, although not significant, for HU308 and the endocannabinoids (Fig. 4e−j, Supplementary Table 6 and 7). These compounds are more lipophilic than LEI-102 and APD371, making them more suitable to traverse the membrane to enter between TM1 and TM7. Notably, HU308 and anandamide also showed a substantially longer ET in our assays compared to the other agonists (Supplementary Table 2). This might suggest a possible relationship between a slower association and membrane channel entry at the CB_2_R. Likewise, for a peptide GPCR a trend in reduced association rate was found with increasing lipophilicity^52^. Nevertheless, this is in contrast with the mechanism at the α_2_-adrenoceptor at which lipophilic compounds had a faster association rate^53^. This shows the diversity in drug-target binding kinetics as receptor-specific properties and thus the importance of investigating these mechanisms for individual receptors^54^.

The discovery of a membrane access channel for endocannabinoids on the CB_2_R is also intriguing from a physiological perspective. Endocannabinoids are produced on demand and act as autocrine or paracrine effectors in the immune system regulating the migration of CB_2_R-expressing immune cells^17^. Our results suggest that endocannabinoids first have to travel through the plasma membrane via lateral diffusion to reach the receptor. This may suggest that the trafficking and cellular uptake of endocannabinoids could be regulated through extracellular or intracellular vesicles that merge with the plasma membrane. Regardless of the exact mechanism of endocannabinoid trafficking, this study provides experimental evidence of a membrane channel located between TM1 and TM7 in CB_2_R that is being used by the endocannabinoids to enter the receptor.

The ligands of the CB_2_R, such as the phytocannabinoids and endocannabinoids, are typically very lipophilic, which comes at a cost of reduced solubility, increased off-target activity, and poor pharmacokinetic properties^10,25^. Thus, balancing lipophilicity of a drug candidate is an important goal in medicinal chemistry. The first generation of experimental drugs targeting the CB_2_R mimicked the plant-based cannabinoids. Consequently, they were highly lipophilic and suffered from poor clinical translation^10^. New generations of CB_2_R agonists have optimized physico-chemical properties. For instance, LEI-102 and APD371 are orders of magnitude more hydrophilic than HU308. Remarkably, they can bind the same binding pocket in CB_2_R as HU308. Our data revealed that LEI-102 and APD371 do not enter the receptor via the membrane channel like HU308, but gain access most likely via the extracellular space. LEI-102 and APD371 also form a specific (indirect) polar interaction network with H95^2.65^ to activate CB_2_R, which is not observed for HU308. This flexibility of the CB_2_R binding pocket to be activated by a diverse set of chemotypes allows to select for a chemotype with more drug-like properties. This notion is supported by the oral efficacy of LEI-102 in the chemotherapy-induced nephropathy model and lack of CNS-adverse side effects (Figs. 5 and 6).

Targeting CB_2_R with agonists is a promising avenue for the treatment of autoimmune diseases, neuroinflammation, and various forms of tissue injury/inflammation/fibrosis in the liver, heart, brain, and kidney^17^. In this study, we show that LEI-102 protects against cisplatin-induced nephropathy in a CB_2_R-dependent manner by attenuating kidney inflammation and injury (Fig. 5). We also show that CB_2_R knockout mice develop more severe nephropathy compared to their wild types suggesting a protective role of endocannabinoid-CB_2_R signaling during kidney injury. These results are consistent with protective effects of CB_2_R agonists in various models of kidney injury/diseases and deleterious effect of CB_2_R deletion in these models^29,55–63^.

In conclusion, we have discovered LEI-102 as a selective CB_2_R agonist that is efficacious in attenuating tissue injury in chemotherapy-induced nephropathy model without inducing CNS-mediated side effects. Using LEI-102 and five other CBR agonists, we have shown that the physicochemical properties determine not only pharmacokinetic properties of ligands, but also how they engage with their target. Altogether, we elucidated several important molecular mechanisms for selective engagement and activation of the CB_2_R, which may have implications for drug design and lipid signaling at GPCRs in general.

## Methods

### General materials for functional assays

Monoclonal M2 mouse anti-FLAG primary antibody (#F3165) was purchased from Sigma-Aldrich (Zwijndrecht, the Netherlands), while secondary goat anti-mouse HRP-conjugated antibody (#115-035-003) was bought from Jackson ImmunoResearch Laboratories (West Grove, PA, USA). Bicinchoninic acid (BCA) ad BCA protein assay reagent was obtained from Pierce Chemical Company (Rockford, IL, USA). [^3^H]RO6957022 (specific activity 82.83 Ci mmol^−^^1^) was custom synthesized at F. Hoffman-La Roche Ltd (Basel, Switzerland). [^35^S]GTPγS (specific activity 1250 Ci mmol^−1^ #NEG030H250UC), [^3^H]CP55,940 (specific activity 108.5 Ci mmol^−1^ #NET1051250UC) and GF/C filter plates (#6055690) were purchased from PerkinElmer (Waltham, MA, USA). CP55,940 (#C1112), AM630 (#SML0327) and DL-dithiotreitol (DTT, #646563) were obtained from Sigma-Aldrich, HU308 (#H800010) was from LKT Laboratories (St. Paul, MN, USA), APD371 was provided by F. Hoffmann-La Roche Ltd, anandamide (AEA, #1339), 2-Arachidonylglycerol (2-AG, #1298) and phenylmethylsulfonyl fluoride (PMSF, #4486) were purchased from Tocris Bioscience (Bristol, UK) and GDP (#J61646) was from Thermo Fisher Scientific (Waltham, MA, USA). All buffers and solutions were prepared using Millipore water (deionized using a MilliQ A10 Biocel with a 0.22 µm filter) and analytical grade reagents and solvents. Buffers are prepared at room temperature (RT) and stored at 4 °C, unless stated otherwise.

### Cell lines

*Spodoptera frugiperda* (*Sf* 9) cells were used for CB_2_R-G_i_ co-expression for cryo-EM studies. *Sf *9 cells were grown in ESF 921 medium (Expression systems) at 27 °C and 125 rpm. For transfections, human embryonic kidney 293T (HEK293T; female, ATCC #CRL-3216) cells were grown as monolayers in culture medium i.e. Dulbecco’s Modified Eagle’s Medium (Sigma-Aldrich #6546), supplemented with 10% fetal calf serum (Sigma-Aldrich #F7524), 2 mM L-glutamine (Sigma-Aldrich #G8541), 100 IU/mL penicillin and 100 µg/mL streptomycin (Duchefa Biochemie #P0142 and #S0148) under a humidified atmosphere at 37 °C with 5% CO_2_. Subculture was done twice a week at 80–90% confluence on 10 cm ø plates by trypsinization. CHO cells stably expressing hCB_2_R (CHOK1_hCB_2_bgal; PathHunter EA Parental Cell line, female, DiscoverX #93-0706C2) were cultured in Ham’s F12 Nutrient Mixture (Sigma-Aldrich #4888) supplemented with 10% fetal calf serum, 2 mM L-glutamine, 100 IU/mL penicillin, 100 µg/mL streptomycin, 300 µg/mL hygromycin (Bio-Connect #ANT-HG-5) and 800 µg/mL G418 (Bio-Connect #SC-29065B) in a humidified atmosphere at 37 °C with 5% CO_2_. Cells were subcultured twice a week when reaching 80–90% confluence on 10 or 15 cm ø plates by trypsinization.

### Synthesis of LEI-102

All reagents and solvents were purchased from commercial sources and were of analytical grade (Sigma-Aldrich, BroadPharm®). Reagents and solvents were not further purified before use. All moisture sensitive reactions were performed under inert atmosphere. Solvents were dried using 4 Å molecular sieves prior to use when anhydrous conditions were required. Water used in reactions was always demineralized. Analytical Thin-layer Chromatography (TLC) was routinely performed to monitor the progression of a reaction and was conducted on Merck Silica gel 60 F254 plates. Reaction compounds on the TLC plates were visualized by UV irradiation (λ_254_) and/or spraying with potassium permanganate solution (K_2_CO_3_ (40 g), KMnO_4_ (6 g), and H_2_O (600 mL)), ninhydrin solution (ninhydrin (1.5 g), n-butanol (100 mL) and acetic acid (3.0 mL)) or molybdenum solution ((NH_4_)_6_MO_7_ · 4 H_2_O (25 g/L) and (NH_4_)_4_Ce(SO_4_)4 · H_2_O (10 g/L) in sulfuric acid (10%)) followed by heating as appropriate. Purification by flash column chromatography was performed using Screening Devices B.V. silica gel 60 (40–63 µm, pore diameter of 60 Å). Solutions were concentrated using a Heidolph laborata W8 4000 efficient rotary evaporator with a Laboport vacuum pump.

Analytical purity was determined with Liquid Chromatography-Mass Spectrometry (LC-MS) using a Finnigan LCQ Advantage MAX apparatus with electrospray ionization (ESI), equipped with a Phenomenex Gemini 3 μm NX-C18 110 Å column (50 × 4.6 mm), measuring absorbance at 254 nm using a Waters 2998 PDA UV detector and the m/z ratio by using an Acquity Single Quad (Q1) detector. Injection was with the Finnigan Surveyor Autosampler Plus and pumped through the column with the Finnigan Surveyor LC pump plus to be analyzed with the Finnigan Surveyor PDA plus detector. Samples were analyzed using eluent gradient 10% → 90% ACN in MilliQ water (+0.1% TFA (v/v)).

For purification by mass guided preparative High-Performance Liquid Chromatography (Prep-HPLC) the Waters AutoPurification HPLC/MS apparatus was used with a Gemini prep column 5 μm 18 C 110 Å (150 × 21.2 mm), Waters 2767 Sample manager, Waters 2545 Binary gradient module, Waters SFO System fluidics organizer, Waters 515 HPLC pump M, Waters 515 HPLC pump L attached to a Waters SQ detector Acquity Ultra performance LC.

^1^H, ^13^C, ^1^H-COSY and HSQC Nuclear Magnetic Resonance (NMR) spectra were recorded on a Bruker AV 300 (300/75 MHz), AV 400 (400/100 MHz) or AV 500 (500/125 MHz) spectrometer at ambient temperature using CDCl_3_ as solvent. Chemical shifts (δ) are referenced in parts per million (ppm) with tetramethylsilane (TMS) or CDCl_3_ resonance as the internal standard peak (CDCl_3_/TMS, δ 0.00 for ^1^H (TMS), δ 77.16 for ^13^C (CDCl_3_)). Multiplicity is reported as s = singlet, d = doublet, dd = doublet of doublet, t = triplet, q = quartet, p = quintet, m = multiplet. Coupling-constants (*J*) are reported in Hertz (Hz) (Supplementary Fig. 1)

### (6-bromo-3-fluoropyridin-2-yl)methanol (2)

To a solution of 6-bromo-3-fluoro-2-methylpyridine (**1**, 10.7 g, 56.3 mmol, 1 eq) under an inert atmosphere at 0 °C in DCM (370 mL) was added portion-wise *m*-CPBA (23.6 g, 70–75%, 100 mmol, 1.8 eq). The reaction mixture was stirred at room temperature (rt) for 4 days. Sat. NaHCO_3_ and sat. Na_2_S_2_O_3_ was added (1:1, v/v) and the layers were separated. The aqueous layer was extracted thrice with DCM. The combined organic layer was dried over MgSO_4_, filtered, and concentrated under reduced pressure. To the residue was added TFAA (17 mL, 122 mmol, 2.2 eq) at 0 °C. After 15 min, the temperature was increased to 55 °C for 3 h. The mixture was concentrated under reduced pressure, redissolved in DCM and sat. Na_2_CO_3_ was added. The layers were separated and the organic layer was washed with sat. NaHCO_3_. The solvent was evaporated and the crude was dissolved in THF:MeOH (20:1, v/v) and K_2_CO_3_ (18.2 g, 132 mmol, 2.3 eq) was added. After 17 h H_2_O was added and the layers were separated. The aqueous layer was extracted thrice with EtOAc. The combined organic layers were dried over MgSO_4_, filtered, and the solvent evaporated under reduced pressure. The crude was purified with flash column chromatography (10–20% EtOAc in pentane) to yield 5.79 g (19.7 mmol, 35%) of a white solid. ^1^H‑NMR (500 MHz, CDCl_3_) δ 7.42 (ddt, *J* = 8.5, 3.5, 0.7 Hz, 1H), 7.29 (t, *J* = 8.5 Hz, 1H), 4.80 (d, *J* = 3.3 Hz, 2H). ^13^C-NMR (126 MHz, CDCl_3_) δ 156.10 (d, *J* = 256.2 Hz), 148.74 (d, *J* = 19.1 Hz), 135.01 (d, *J* = 2.9 Hz), 128.17 (d, *J* = 4.2 Hz), 126.09 (d, *J* = 19.8 Hz), 59.07.

### (6-bromo-3-fluoropyridin-2-yl)methyl methanesulfonate (3)

To a cooled (0 °C) mixture of (6-bromo-3-fluoropyridin-2-yl)methanol (1.6 g, 7.8 mmol, 1 eq) and Et_3_N (2.5 mL, 17.9 mmol, 2.3 eq) in dry THF (40 mL) was added dropwise MsCl (1.0 mL, 12.9 mmol, 1.7 eq). After stirring at rt for 1 h the solution was concentrated under reduced pressure. DCM and H_2_O were added and the layers were separated. The aqueous layer was extracted thrice with DCM. The combined organic layers were washed with brine, dried over MgSO_4_, filtered, and the solvent evaporated under reduced pressure to yield 1.65 g (5.8 mmol, 75%) of an yellow solid. ^1^H‑NMR (500 MHz, CDCl_3_) δ 7.52 (dd, *J* = 8.6, 3.5 Hz, 1H), 7.37 (t, *J* = 8.5 Hz, 1H), 5.33 (d, *J* = 2.1 Hz, 2H), 3.13 (s, 3H). ^13^C‑NMR (126 MHz, CDCl_3_) δ 157.82 (d, *J* = 261.3 Hz), 142.15 (d, *J* = 16.0 Hz), 130.74 (d, *J* = 4.4 Hz), 127.06 (d, *J* = 20.4 Hz), 65.50 (d, *J* = 1.6 Hz), 38.39.

### *N*-((6-bromo-3-fluoropyridin-2-yl)methyl)tetrahydro-2*H*-pyran-4-amine (4)

(6-Bromo-3-fluoropyridin-2-yl)methyl methanesulfonate (1.49 g, 5.3 mmol, 1 eq), K_2_CO_3_ (1.6 g, 11.6 mmol, 2.2 eq) and tetrahydro-2*H*-pyran-4-amine (0.66 mL, 6.7 mmol, 1.3 eq) were suspended in acetonitrile and stirred at 50 °C for 6 h, then an additional 3 days at rt. After dilution with DCM and H_2_O the layers were separated. The aqueous layer was extracted thrice with DCM. The combined organic layers were dried over MgSO_4_, filtered, and the solution evaporated under reduced pressure. The crude was purified with flash column chromatography (20–100% EtOAc in pentane) to yield 1.01 g (3.5 mmol, 67%) as a yellow oil. ^1^H‑NMR (300 MHz, CDCl_3_) δ 7.40 (dd, *J* = 8.6, 3.6 Hz, 1H), 7.35–7.26 (m, 1H), 4.08–3.95 (m, 4H), 3.42 (td, *J* = 11.6, 2.2 Hz, 2H), 2.74 (tt, *J* = 10.5, 4.1 Hz 1H), 1.89 (ddd, *J* = 12.7, 4.5, 2.3 Hz, 2H), 1.52 (dtd, *J* = 13.1, 11.0, 4.5 Hz, 2H). ^13^C‑NMR (75 MHz, CDCl_3_) δ 157.12 (d, *J* = 255.9 Hz), 149.21 (d, *J* = 17.0 Hz), 127.83 (d, *J* = 4.2 Hz), 125.97 (d, *J* = 21.2 Hz), 66.76, 53.64, 44.90, 33.59.

### 2-((4-bromobenzyl)amino)acetamide (6)

To a mixture of 4-bromobenzaldehyde (**5**, 9.2 g (49.7 mmol, 1.1 eq) and 2‑aminoacetamide hydrochloride (5.06 g, 45.8 mmol, 1.0 eq) in MeOH:H_2_O (170 mL, 5:1, v/v) was added NaOH (2.06 g, 51.5 mmol, 1.1 eq) and left to stir at rt overnight. NaBH_4_ (3.6 g, 95.2 mmol, 2.1 eq) was added and the solution was stirred overnight at rt. The solution was acidified to pH 3 with 2 M HCl, then neutralized with sat. aqueous NaHCO_3_. Methanol was evaporated under reduced pressure and the resulting slurry was filtered to yield 11.0 g (45.2 mmol, 91%) of a white solid. ^1^H‑NMR (300 MHz, methanol-d_4_) δ 7.69–7.59 (m, 2H), 7.47–7.38 (m, 2H), 4.22 (s, 2H), 3.81 (s, 2H).

### 1-(4-bromobenzyl)imidazolidine-2,4-dione (7)

To a suspension of 2-((4-bromobenzyl)-amino)acetamide (10.0 g, 40.1 mmol, 1,0 eq) in acetonitrile (300 mL) were added CDI (13.86 g, 85.5 mmol, 2.1 eq) and DMAP (10.2 g, 83.5 mmol, 2.1 eq). The mixture was heated to 60 °C under inert atmosphere for 70 h. HCl (1 M, 250 mL) was added and the aqueous layer extracted thrice with EtOAc. The combined organic layers were washed with H_2_O and brine, dried over MgSO_4_, filtered, and the solvent evaporated under reduced pressure. The crude was purified with flash column chromatography with dry loading over Celite (5-10% acetone in DCM) to yield 3.95 g (14.7 mmol, 37%) of a yellow solid. ^1^H‑NMR (300 MHz, CDCl_3_) δ 7.83 (bs, 1H), 7.56 – 7.45 (m, 2H), 7.20 – 7.10 (m, 2H), 4.49 (s, 2H), 3.79 (s, 2H). ^13^C‑NMR (75 MHz, CDCl_3_) δ 132.41, 129.95, 77.58, 77.16, 76.74, 50.36, 46.01.

### 1-(4-bromobenzyl)-3-isobutylimidazolidine-2,4-dione (8)

To solution of 1-(4-bromobenzyl)imidazolidine-2,4-dione (2.00 g, 7.4 mmol, 1,0 eq) in anhydrous DMF (18 mL) were subsequently added K_2_CO_3_ (3.08 g, 22.3 mmol, 3,0 eq) and 1-bromo-2-methylpropane (1.62 mL, 14.9 mmol, 2,0 eq) and the mixture was stirred for 20 h at rt. The mixture was filtered and the filtrate diluted with diethyl ether and washed thrice with water (3 × 50 mL). The combined organic layers were washed with brine, dried (MgSO_4_), filtered, and concentrated under reduced pressure. The crude was purified with flash column chromatography (10–40% EtOAc in pentane) to yield 2.12 g (6.52 mmol, 88%) of a white solid. LCMS (LCQ Fleet, 10–90%): *t*_r_ = 7.00 min, m/z: 325.17 [M + H]^+^, 327.08 [M + H]^+^ (Br). ^1^H-NMR (300 MHz, CDCl_3_) δ 7.47 (d, *J* = 8.3 Hz, 2H), 7.14 (d, *J* = 8.3 Hz, 2H), 4.52 (s, 2H), 3.74 (s, 2H), 3.33 (d, *J* = 7.4 Hz, 2H), 2.15–2.04 (m, 1H), 0.91 (d, *J* = 6.8 Hz, 6H). ^13^C NMR (75 MHz, CDCl_3_) δ 169.57, 156.78, 134.41, 131.79, 129.48, 121.77, 60.01, 48.61, 45.98, 45.71, 28.57, 19.70.

### 3-isobutyl-1-(4-(4,4,5,5-tetramethyl-1,3,2-dioxaborolan-2-yl)benzyl)imidazolidine-2,4-dione (9)

A mixture of 1-(4-bromobenzyl)-3-isobutylimidazolidine-2,4-dione (0.50 g, 1.54 mmol, 1 eq), KOAc (0.66 g, 6.76 mmol, 4.4 eq) and bis(pinacolato)diboron (0.59 g, 2.31 mmol, 1.5 eq) in DMF (10 mL) was sonicated for 15 min under argon flow. Subsequently, Pd(dppf)Cl_2_ (0.07 g, 0.09 mmol, 0.06 eq) was added and the mixture was stirred at 75 °C for 20 h. The mixture was cooled to rt, diluted with EtOAc (100 mL) and water (10 mL) and the layers were separated. The water layer was extracted thrice with EtOAc (3 × 20 mL). The combined organic layers were extracted with sat. aqueous NaHCO_3_, water and brine, dried (MgSO_4_), filtered, and concentrated under reduced pressure. The raw product was co-evaporated with CHCl_3_ and used in the next step without further purification.

### 1-(4-(5-fluoro-6-(((tetrahydro-2*H*-pyran-4-yl)amino)methyl)pyridin-2-yl)benzyl)-3-isobutylimidazolidine-2,4-dione (LEI-102)

To a degassed mixture of *N*-((6-bromo-3-fluoropyridin-2-yl)methyl)tetrahydro-2*H*-pyran-4-amine (**4**, 0.29 g, 1.0 mmol, 1,0 eq), 3-isobutyl-1-(4-(4,4,5,5-tetramethyl-1,3,2-dioxaborolan-2-yl)benzyl)imidazolidine-2,4-dione (**9**, 0.56 g, ~1.5 mmol, crude) and K_2_CO_3_ (1.29 g, 6.0 mmol, 6,0 eq) in toluene:ethanol (10 mL, 4:1, v/v) was added under argon atmosphere Pd(PPh_3_)_4_ (0.18 g, 0.10 mmol, 0.1 eq). The resulting mixture was stirred for 18 h at 75 °C, subsequently cooled to rt, and filtered. The filtrate was diluted with EtOAc and washed with water and brine, dried (MgSO_4_), filtered, and concentrated under reduced pressure. The crude was purified with flash column chromatography (0‑20% MeOH in EtOAc) to yield 0.24 g of a white solid (0.53 mmol, 53%). Further purification with preparative HPLC resulted in a yield of 0.204 g (0.45 mmol, 45%). LCMS (LCQ Advantage, 10–90%): *t*_*r*_ = 5.32 min, m/z: 455.27 [M + H]^+^, 908.93 [2 M + H]^+^. HRMS (ESI+) m/z: calcd. for C_25_H_32_FN_4_O_3_ [M + H], 455.245; found, 455.245. ^1^H NMR (400 MHz, CD_3_CN) δ 8.05 (d, *J* = 8.3 Hz, 2H), 7.86 (dd, *J* = 8.7, 3.6 Hz, 1H), 7.61 (t, *J* = 9.0 Hz, 1H), 7.34 (d, *J* = 8.1 Hz, 2H), 4.53 (s, 2H), 4.45 (s, 2H), 3.93 (dd, J = 11.4, 4.4 Hz, 2H), 3.77 (s, 1H), 3.47 (tt, *J* = 11.8, 3.8 Hz, 2H), 3.30 (td, *J* = 11.9, 1.9 Hz, 2H), 3.24 (d, *J* = 7.3 Hz, 2H), 2.05 (br d, *J* = 13.3 Hz, 2H), 1.99 (dt, *J* = 13.2, 6.6 Hz, 1H), 1.83 (qd, *J* = 12.1, 4.5 Hz, 2H), 0.88 (d, *J* = 6.7 Hz, 6H). ^13^C NMR (100 MHz, CD_3_CN) δ 171.54, 157.25 (d, *J* = 226.6 Hz), 156.12, 153.03 (d, *J* = 4.5 Hz), 140.51 (d, *J* = 16.1 Hz), 138.83, 137.70, 129.13, 128.22, 125.56 (d, *J* = 18.8 Hz), 122.81 (d, *J* = 4.3 Hz), 118.38, 66.55, 55.55, 50.30, 46.81 (d, *J* = 7.9 Hz), 42.95, 30.02, 28.32, 20.32.

### Constructs

The N-BRIL fused wild type (WT) human CB_2_R construction and co-expression of G protein for cryo-EM study were performed using the similar procedure as described before^34^. In brief, the WT human CB_2_R was modified to contain a fusion protein BRIL to improve the protein expression and thermostability, along with a 10×His-tag and a FLAG-tag at the N-terminal. The CB_2_R, Gα_i1_ and Gβ_1_γ_2_ subunits were cloned into the pFastBac vector separately using cloning kits.

### Expression and purification of CB_2_R-G_i_-Scfv16 complexes

Methods of complex expression and purification in the current study have been described previously^34^. The CB_2_R and G_i_ heterotrimer were co-expressed in *Sf *9 insect cells using the Bac-to-Bac Baculovirus Expression System (Invitrogen). Cells were infected with three separate virus preparations for CB_2_R, Gα_i1_ and Gβ_1_γ_2_ at a ratio of 1:2:2 at a cell density of 2.5 × 10^6^ cells/mL. After 48 h, the cell culture was collected by centrifugation and the cell pellets were stored at −80 °C until use. The cell pellets were thawed and lysed in the hypotonic buffer of 10 mM HEPES (pH 7.5), 10 mM MgCl_2_, 20 mM KCl with EDTA-free complete protease inhibitor cocktail tablets (Roche, #5056489001). The CB_2_R-G_i_ complex was formed in membranes by addition of 25 μM agonist (LEI-102, APD371, HU308, and CP55,940, respectively) and 2 units of apyrase (NEB, #M0398S) in the presence 500 µg scFv16. The lysate was incubated for overnight at 4 °C and discard the supernatant by centrifugation at 186,000 × *g* for 30 min. Subsequently, the solubilization buffer containing 50 mM HEPES (pH 7.5), 100 mM NaCl, 0.75% (w/v) lauryl maltose neopentyl glycol (LMNG, Anatrace, #4216588), 0.15% (w/v) cholesterol hemisucinate (CHS, Sigma-Aldrich, #C6512) supplemented with 25 μM agonist and 2 units of apyrase (NEB) were added to solubilize complexes for 2 h at 4 °C. Insoluble material was removed by centrifugation at 186,000 × *g* for 30 min and the supernatant was immobilized by batch binding to TALON IMAC resin (Clontech, #635507) including 20 mM imidazole over 6 h at 4 °C. Then, the resin was packed and washed with 15 column volumes (CVs) of washing buffer I containing 25 mM HEPES (pH 7.5), 100 mM NaCl, 10% (v/v) glycerol, 0.1% (w/v) LMNG, 0.02% (w/v) CHS, 30 mM imidazole and 20 μM agonist, and 15 CVs of washing buffer II containing 25 mM HEPES (pH 7.5), 100 mM NaCl, 10% (v/v) glycerol, 0.03% (w/v) LMNG, 0.006% (w/v) CHS, 50 mM imidazole and 20 μM agonist. After that, the protein was eluted using 3 CVs of elution buffer containing 25 mM HEPES (pH 7.5), 100 mM NaCl, 10% (v/v) glycerol, 0.01% (w/v) LMNG, 0.002% (w/v) CHS, 250 mM imidazole and 25 μM agonist. Finally, the complex was concentrated using the centrifugal filter with 100 kDa molecular weight cutoff and loaded onto a Superdex200 10/300 GL column (GE Healthcare) with buffer containing 20 mM HEPES (pH 7.5), 100 mM NaCl, 0.00075% (w/v) LMNG, 0.00025% GDN (Anatrace, #GDN101), 0.0001% (w/v) CHS, 100 μM TCEP. The fractions consisting of purified CB_2_R-G_i_ complex were collected and concentrated to 0.8–1.0 mg/mL for electron microscopy experiments.

### Cryo-EM grid preparation and data collection

For cryo-EM grids preparation of the CB_2_R-G_i_ complexes, 3 μL of the concentrated protein was loaded to a glow-discharged holey carbon grid (CryoMatrix Amorphous alloy film R1.2/1.3, 300 mesh), and subsequently were plunge-frozen in liquid ethane using a Vitrobot Mark IV (Thermo Fisher Scientific). The chamber of Vitrobot was set to 100% humidity at 4 °C. The sample was blotted for 2.5 s with blot force 2. Cryo-EM images were collected on a Titan Krios microscope operated at 300 kV equipped with a Gatan Quantum energy filter, with a slit width of 20 eV, a Gatan K2 summit direct electron camera (Gatan). Images were taken at a dose rate of 8e^−^/Å^2^/s with a defocus range of −0.8 to −2.0 μm using SerialEM software^64^ in EFTEM nanoprobe mode, with 50 μm C2 aperture, at a calibrated magnification of 130,000 corresponding to a magnified pixel size of 1.04 Å. The total exposure time was 8.1 s and 45 frames were recorded per micrograph.

### Cryo-EM image processing

The cryo-EM data processing was performed with CryoSPARC^65^. For CB_2_R-Gα_i_-scFv16-APD371/LEI-102/HU308/CP55,940 dataset, a total of 7443, 5282, 7530, and 6473 movies were collected, respectively. For all datasets, patch motion correction was used for beam-induced motion correction. Contrast transfer function (CTF) parameters for each micrograph were determined by patch CTF estimation. Using Blob Picker in CryoSPARC to auto pick particles in the first 500 micrographs of CB_2_R-Gα_i_-scFv16-APD371 complex dataset and then 258,347 particles were extracted to conduct 2D classification. 9277 particles in good 2D patterns were selected as templates to pick better particles. 5,239,870, 3,398,611, 4,653,294, and 3,595,875 particles extracted, respectively, in a 256 Å box were divided into three hundred two-dimensional (2D) class averages with a maximum alignment resolution of 6 Å. Then, 1,152,146, 762,471, 355,832, and 440,292 particles were selected from good 2D classification after two round 2D classification, individually. Following 2D classification, these particles were subjected for ab initio reconstruction into four classes. After heterogeneous refinement, homogeneous refinement, non-uniform refinement and local refinement of the best-looking dataset in CryoSPARC, the final map has an indicated global resolution of 3.08, 2.98, 2.97, and 2.84 Å at a Fourier shell correlation (FSC) of 0.143, respectively. Local resolution was determined using the Bsoft package with half maps as input maps^66^.

### Model building and refinement

For CB_2_R-G_i_-scFv16 complex, the CB_2_R-AM12033 cryo-EM structure and G_i_ protein in CB_2_R were used as the starting model. The model was docked into the EM density map using Chimera^67^, followed by iterative manual adjustment and rebuilding in COOT^68^ and phenix.real_space_refine in Phenix^69^. The model statistics were validated using MolProbity^70^. Structural figures were prepared in Chimera and PyMOL (http://www.pymol.org). The final refinement statistics were provided in Supplementary Table 4. The extent of any model overfitting during refinement was measured by refining the final model against one of the half-maps and by comparing the resulting map versus model FSC curves with the two half-maps and full model.

### Generation of mutants

The WT CB_1_R and CB_2_R genes were subcloned into vector pcDNA3.1 with an N-terminal HA signal peptide and FLAG-tag. Mutations were introduced by QuikChange PCR (as described by supplier).

### Transfection

24 h prior to transfection, HEK293T cells were seeded on 10 cm ø plates to reach approximately 50% confluence at the start of transfection. The cells were transfected with 10 µg plasmid DNA of WT hCB_2_R or hCB_1_R receptor, or mutant receptor using the calcium phosphate precipitation method^71^. In short, a DNA-calcium mix was made containing 270 mM CaCl_2_ and 10 µg plasmid DNA to which Hank’s Balanced Salt Solution (HBSS; 280 mM NaCl, 10 mM KCl, 1.5 mM Na_2_HPO_4_, and 50 mM HEPES) was added in a 1:1 (v/v) ratio and mixed by aeration to create consistent calcium phosphate precipitates. For transfection, 1 mL DNA-calcium mix was added per 10 cm ø plate, followed by a 48 h incubation under a humidified atmosphere at 37 °C with 5% CO_2_.

### Enzyme-linked immunosorbent assay (ELISA)

Receptor expression after transfection was measured in an enzyme-linked immunosorbent assay (ELISA). After 24 h of transfection, HEK293T cells were detached with phosphate-buffered saline (PBS)/EDTA and seeded into a sterile 96-well poly-D-lysine coated plate at a density of 100,000 cells per well and kept under a humidified atmosphere at 37 °C with 5% CO_2_. After an additional 24 h, cells were washed with PBS and fixed with 4% formaldehyde for 10 min at room temperature (rt). Cells were washed twice with Tris-buffered saline (TBS) and were blocked with TBS supplemented with 0.1% TWEEN 20 (TBST) and 2% BSA (w/v)) for 30 min at rt while shaking. Subsequently, the cells were incubated with monoclonal M2 mouse anti-FLAG primary antibody (1:4000) for 2 h at rt while shaking. After removal of the antibody, the cells were washed three times with TBST and incubated with the secondary goat anti-mouse HRP-conjugated antibody (1:10,000) for 1 h at rt while shaking. After a final wash with TBS, the cells were treated with 3,3’,5,5’-Tetramethylbenzidine (TMB, Sigma-Aldrich #T0440) in the dark for maximally 10 min at rt to visualize immunoreactivity. The reaction was quenched with 1 M H_3_PO_4,_ and absorbance was read at 450 nm with a Wallac EnVision 2104 Multilabel reader (PerkinElmer).

### Membrane preparation

For membrane preparation, HEK293T cells were harvested 48 h after transfection. Cells were detached by scraping into 3 mL of PBS and subsequently centrifuged at 2000 × *g* for 5 min. Pellets were resuspended in ice-cold Tris buffer (50 mM Tris-HCl, pH 7.4) and homogenized with an Ultra Turrax homogenizer (IKA-Werke GmbH & Co. KG, Staufen, Germany). Cytosolic and membrane fractions were separated using a high-speed centrifugation step of 31,000 rpm in a Beckman Optima LE-80K ultracentrifuge with Ti70 Rotor for 20 min at 4 °C. After a second cycle of homogenization and centrifugation, the final pellets were resuspended in 50 mM Tris-HCl pH 7.4, 5 mM MgCl_2_ and stored in 100 µL aliquots at −80 °C until use. CHOK1_hCB_2_bgal cells were harvested when reaching 90% confluence in 15 cm ø plates after one week subculture at a 1:6 ratio. Membrane preparation followed a similar procedure as described above. Final membrane pellets were resuspended in 50 mM Tris-HCl pH 7.4 and stored in 100 µL aliquots at −80 °C until use. Membrane protein concentrations were determined using a BCA protein determination assay as described by the manufacturer^72^.

### [^3^H]RO6957022 competition association assays

For assessment of kinetic agonist binding at hCB_2_R, [^3^H]RO6957022 competition association assays were executed. These assays were previously described with the main difference of incubation at 25 °C compared to 10 °C for identification of more distinct kinetic differences^73^. In short, prior to kinetic assessment of agonist binding, the affinity (IC_50_) of the agonists at the hCB_2_R was determined in [^3^H]RO6957022 displacement assays. CHOK1_hCB_2_bgal were thawed, homogenized, and subsequently diluted to 1 µg protein per well. When studying endocannabinoids, membranes were preincubated with 50 µM PMSF for 30 min. Membranes were incubated with ~1.5 nM [^3^H]RO6957022 and six increasing concentrations of competing agonists in a total volume of 100 µL assay buffer (50 mM Tris-HCl (pH 7.4), 0.1% (w/v) BSA). Incubations were done for 2 h at 10 °C to reach equilibrium. Subsequently, in competition association assays, agonists were incubated at their IC_50_ concentration in the presence of ~1.5 nM [^3^H]RO6957022 in a total volume of 100 µL assay buffer at 10 °C. Competition was initiated by addition of membrane homogenates at different time points for 2 h. Nonspecific binding (NSB) was determined with 10 µM AM630 and organic solvent (DMSO or acetonitrile) concentrations were <1% in all samples. Total radioligand binding (TB) did not exceed 10% of the amount added to prevent ligand depletion. Incubations were terminated by rapid vacuum filtration with ice-cold 50 mM Tris-HCl (pH 7.4), 0.1% (w/v) BSA buffer through Whatman GF/C filters using a Filtermate 96-well harvester (PerkinElmer). Filters were dried for at least 30 min at 55 °C, and subsequently 25 µL MicroScint scintillation cocktail (PerkinElmer #6013621) was added per well. Filter-bound radioactivity was measured by scintillation spectrometry using a Microbeta^2^ 2450 counter (PerkinElmer).

### [^35^S]GTPγS binding assays

G protein activation by agonists LEI-102, APD371, HU308, CP55,940 AEA, and 2-AG was measured by binding of radiolabeled [^35^S]GTPγS to the cannabinoid receptors as previously described^25^. In short, transient HEK293T membrane homogenates (10 µg/well) were diluted in assay buffer (50 mM Tris-HCl (pH 7.4), 5 mM MgCl_2_, 150 mM NaCl, 1 mM EDTA, 0.05% BSA (w/v) and 1 mM DTT, freshly prepared every day) and were pretreated with 10 µg saponin and 1 µM GDP. For endocannabinoid samples, the membranes were additionally pretreated for 30 min with 50 µM PMSF before agonist addition. To determine the G protein activation, the membranes were incubated with 10 µM or six increasing concentrations of agonist (ranging from 0.01 nM to 10 µM) for 30 min at rt. Basal receptor activity was determined in the presence of vehicle only (0.2% DMSO/acetonitrile). [^35^S]GTPγS (0.3 nM) was added and the mixture was co-incubated for an additional 90 min at 25 °C while shaking at 400 rpm. Filtration was performed, and filter-bound radioactivity was determined as described under [^3^H]RO6957022 Competition Association Assays except for using ice-cold 50 mM Tris-HCl (pH 7.4), 5 mM MgCl_2_ buffer.

### [^3^H]CP55,940 homologous and heterologous displacement assays

Agonist affinity (K_i_) on WT and mutant receptors was determined in [^3^H]CP55,940 displacement assays. The amount of transient HEK293T membrane, ranging from 0.75 µg to 10 µg protein per well, was chosen to obtain a specific [^3^H]CP55,940 binding window of 1200-1500 disintegrations per minute (dpm) except for the CB_2_R-Quadruple^TM1,7^ mutant, for which a window of ~500 dpm could be obtained using 20 µg protein per well. Membranes were thawed and subsequently homogenized using the Ultra Turrax homogenizer. For the endocannabinoid assays, the membranes were preincubated for 30 min with 50 µM PMSF. Homologous displacement assays were performed with 1.5 nM final concentration [^3^H]CP55,940 and when necessary supplemented with an additional concentration of 0.55 nM [^3^H]CP55,940 in the presence of competing CP55,940 (ranging from 0.01 nM to 1 µM) in assay buffer (50 mM Tris-HCl (pH 7.4), 5 mM MgCl_2_, 0.1% (w/v) BSA). Heterologous displacement assays were executed for LEI-102, APD371, HU308, AEA, and 2-AG using 1.5 nM final concentration [^3^H]CP55,940 with one concentration (10 µM) or six increasing concentrations (ranging from 0.1 nM to 10 µM) in assay buffer. For both assays, binding was initiated by addition of membrane homogenates to reach a final volume of 100 µL. NSB was determined using 10 µM CP55,940 and organic solvent (DMSO or acetonitrile) concentrations were <1% in all samples. TB did not exceed 10% of the amount added to prevent ligand depletion. Incubation was done for 2 h at 25 °C to reach equilibrium. Filtration was performed, and filter-bound radioactivity was determined as described under [^3^H]RO6957022 Competition Association Assays except for using ice-cold 50 mM Tris-HCl (pH 7.4), 5 mM MgCl_2_, 0.1% (w/v) BSA buffer.

### Cisplatin-induced nephropathy

Ten to twelve-week-old male/female *C57BL/6J* mice were obtained from The Jackson Laboratory (Bar Harbor, ME, USA). CB_2_R knockout mice (CB_2_R^−/−^) and their wild-type littermates (CB_2_R^+/+^) were developed as described previously and had been backcrossed to a C57BL/6J background^74^. All animals were kept in a temperature-controlled environment (20–22 °C) with a 12 h light–dark cycle and were always allowed free access to food and water. All animal experiments reported in this manuscript complied with the National Institutes of Health “Guide for the Care and Use of Laboratory Animals” (NIH publication 86–23 revised 1985) and were approved by the Institutional Animal Care and Use Committee of the National Institute on Alcohol Abuse and Alcoholism (Bethesda, MD).

The well-established model of cisplatin-induced nephropathy was used^63^. Mice (CB_2_R^−/−^ and CB_2_R^+/+^) were sacrificed 72 h after a single injection of cisplatin (cis-diamine platinum (II) dichloride (Sigma#P4394) 25 mg/kg i.p.; freshly dissolved in physiological saline) by cervical dislocation under deep anesthesia with 5% isoflurane, for collection of blood and tissue samples. LEI-102 was given i.p. or by oral gavage (p.o.) at 0.3, 3.0, and 10 mg/kg every day, starting 1.5 h before the cisplatin exposure. The drug was dissolved in a vehicle of DMSO:Tween 80:saline, 1:1:18. After administration of LEI‐102, mice were killed by cervical dislocation under deep anesthesia with 5% isoflurane, for collection of blood and tissue samples at the time described in the figure. The tetrad assay in mice has previously been described in detail^29^.

### Biochemistry, histopathology, immunostaining, real-time PCR

Markers of kidney dysfunction (BUN and CREA), histopathology (PAS staining), immunostaining or ELISA for 3-nitrotyrosine (3-NT; Cell Biolabs #STA-305) and 4-hydroxynonenal (4-HNE; Cell Biolabs#STA-838), and real-time PCR (Primers from Qiagen, SYBER Green Vita Scientific#MEIF01301, High-Capacity cDNA Reverse Transcription Kit, Thermo Fisher Scientific#4368813) for inflammatory cytokines were performed as previously described^63^. Tubular damage scores were determined based on the percentage of tubules showing epithelial necrosis where 0= normal; 1, <10%; 2, 10–25%; 3, 26–75%; 4, >75%. Tubular necrosis was defined as the loss of the proximal tubular brush border, blebbing of apical membranes, tubular epithelial cell detachment from the basement membrane, or intraluminal aggregation of cells and proteins. The morphometric examination was performed in a blinded manner. Ten fields were scored from each mouse kidneys at 200× magnification, and average scores were determined for each mouse. For final quantification graph, average tubular damage scores of six mice/group were plotted.

### Quantification and statistical analysis

All experimental data were analyzed using GraphPad Prism 9.0 (GraphPad Software Inc., San Diego, CA). All values obtained are means ± standard error of the mean (SEM) of at least three independent experiments performed in duplicate, unless stated otherwise.

From [^3^H]RO6957022 competition association assays, the *k*_on_ and *k*_off_ were determined by non-linear regression analysis, using the “kinetics of competitive binding” model as described by Motulsky and Mahan (Motulsky and Mahan, 1984):

$${K}_{a}={k}_{1}\cdot \left[L\right]\cdot {10}^{-9}+{k}_{2}$$

$${K}_{b}={k}_{3}\cdot \left[I\right]\cdot {10}^{-9}+{k}_{4}$$

$$S=\sqrt{{({K}_{a}-{K}_{b})}^{2}+4\cdot {k}_{1}\cdot {k}_{3}\cdot \left[L\right]\cdot \left[I\right]\cdot {10}^{-18}}$$

$${K}_{f}=0.5\cdot \left({K}_{a}+{K}_{b}+S\right)$$

$${K}_{s}=0.5\cdot \left({K}_{a}+{K}_{b}-S\right)$$

$$Q=\frac{B\max \cdot {k}_{1}\cdot \left[L\right]\cdot {10}^{-9}}{{K}_{f}-{K}_{s}}$$

$$\left[Y\right]=Q\cdot \left(\frac{{k}_{4}\cdot ({K}_{f}-{K}_{s})}{{K}_{f}\cdot {K}_{s}}+\frac{{k}_{4}-{K}_{f}}{{K}_{f}}\cdot {{{{{{\rm{e}}}}}}}^{\left(-{K}_{f}\cdot X\right)}-\frac{{k}_{4}-{K}_{s}}{{K}_{s}}\cdot {{{{{{\rm{e}}}}}}}^{\left(-{{K}_{S}} \cdot {X}\right)}\right)$$

Where [*L*] is the radioligand concentration per experiment (~1.5 nM), *I* is the IC_50_ concentration of agonist (nM), *X* is the time (s), and *Y* is the specific binding of the radioligand (dpm). *K*_*a*_ and *K*_*b*_ are the observed association rate constants (*k*_obs_) of the radioligand and the agonist of interest, respectively. *k*_*1*_ and *k*_*3*_ are the association rate constants (*k*_on_ in M^−1^s^−1^) of [^3^H]RO6957022 (determined per experiment) and the agonist of interest, respectively. Similarly, *k*_*2*_ and *k*_*4*_ are the dissociation rate constants (*k*_off_ in s^−1^) of [^3^H]RO6957022 (experimentally determined at 4.3 × 10^−4^ s^−1^, data not shown) and the agonist of interest, respectively. The engagement time (ET in seconds) of the agonists of interest was determined at 1 µM of agonist using the equation ET = 1/(*k*_on_ · 1 × 10^−6^). The residence time (RT in min) was calculated using the equation RT = 1/(60 · *k*_off_)^75^. The association and dissociation rate constants were used to calculate the kinetic *K*_D_ using: *K*_D_ = *k*_off_/*k*_on_.

[^35^S]GTPγS agonist responses on hCB_2_R constructs were baseline-corrected for the individual mutant’s basal activity. The responses were normalized to the basal activity of the construct (0%) and top of the CP55,940 (for WT responses only) or WT curve (for mutants, 100%). The potency (pEC_50_) and efficacy (E_max_) values were obtained by non-linear regression to a sigmoidal concentration-effect curve with a Hill slope of 1 by using the “log(agonist) vs response (three parameters)” model. [^35^S]GTPγS data from hCB_1_R constructs were expressed as fold over the mutant’s basal activity to also quantify the effects of CB_2_R selective agonists.

Displacement assays were baseline-corrected with NSB and normalized to this value (0%) and TB (100%). The equilibrium dissociation constants (K_D_) of [^3^H]CP55,940 on different mutants were calculated from homologous displacements by non-linear regression analysis, using the “one-site homologous” model. The half-maximal inhibitory concentrations (pIC_50_) of the agonists in [^3^H]CP55,940 and [^3^H]RO6957022 assays were obtained by non-linear regression analysis of the homologous and heterologous displacement curves and further converted into inhibitory constant pK_i_ using the Cheng-Prusoff equation^76^. In which the experimentally determined K_D_ for each construct was used for [^3^H]CP55,940 assays or 0.78 nM for [^3^H]RO6957022 assays (data not shown).

Differences in pEC_50_, E_max_, pK_D_ and pK_i_ values for each mutant compared to WT were analyzed using a one-way Welch’s ANOVA with Dunnett’s T3 multiple comparisons test or an unpaired Student’s t-test with Welch’s correction. Significant differences are displayed as *p < 0.05; **p < 0.01, ***p < 0.001 and ****p < 0.0001. For the animal experiments all the values are represented as mean ± SEM. Statistical analysis of the data was performed by analysis of variance (ANOVA) followed by Tukey’s post hoc test for multiple comparisons or t-test if appropriate. The analysis was conducted using GraphPad Prism 9 software. *p*  <  0.05 was considered statistically significant.

### Reporting summary

Further information on research design is available in the Nature Portfolio Reporting Summary linked to this article.

## Supplementary information

Supplementary Information

Reporting Summary

## Data Availability

The data that support this study are available from the corresponding authors upon request. The atomic coordinates for CB_2_R-LEI-102-G_i_-scFv16, CB_2_R-APD371-G_i_-scFv16, CB_2_R-HU308-G_i_-scFv16, and CB_2_R-CP55,940-G_i_-scFv16 have been deposited in the Protein Data Bank with the accession codes 8GUT, 8GUQ, 8GUS, and 8GUR. The EM maps for CB_2_R-LEI-102-G_i_-scFv16, CB_2_R-APD371-G_i_-scFv16, CB_2_R-HU308-G_i_-scFv16 and CB_2_R-CP55,940-G_i_-scFv16 have been deposited in EMDB with the codes EMD-34279, EMD-34276, EMD-34278, and EMD-34277, respectively. Source data are provided with this paper.

## Source data

Source Data
