# Supplementary material for: Structural basis of selective cannabinoid CB2 receptor activation
Source: Nat Commun. 2023 Mar 15;14:1447. doi: 10.1038/s41467-023-37112-9 (PMC10017709; doi:10.1038/s41467-023-37112-9)
Supplement: Supplementary file 1 — Supplementary Information [file 41467_2023_37112_MOESM1_ESM.pdf]

Structural basis of selective cannabinoid CB_2 receptor activation

Xiaoting Li[1]*, Hao Chang[1,2]*, Jara Bouma[3]*, Laura V. de Paus[4], Partha Mukhopadhyay[5], Janos Paloczi[5], Mohammed Mustafa[6], Cas van der Horst[3], Sanjay Sunil Kumar[3], Lijie Wu[1,2], Yanan Yu[1,2], Richard J. B. H. N. van den Berg[4], Antonius P.A. Janssen[4], Aron Lichtman[6], Zhi-Jie Liu[1,2#], Pal Pacher[5#], Mario van der Stelt[4#], Laura H. Heitman[3#] and Tian Hua[1,2#]

Affiliations:

[1]iHuman Institute, ShanghaiTech University, Shanghai 201210, China

[2]School of Life Science and Technology, ShanghaiTech University, Shanghai, China

[3]Division of Drug Discovery and Safety, LACDR, Leiden University & Oncode Institute, the Netherlands

[4]Department of Molecular Physiology, LIC, Leiden University & Oncode Institute, the Netherlands

[5]Laboratory of Cardiovascular Physiology and Tissue Injury, National Institute of Health/NIAAA, Rockville, Maryland, USA

[6]Department of Pharmacology and Toxicology, Virginia Commonwealth University, Richmond, VA, USA

*These authors contributed equally

[#]Coorespondence: huatian@shanghaitech.edu.cn (T.H.); l.h.heitman@lacdr.leidenuniv.nl (L.H.H); m.van.der.stelt@chem.leidenuniv.nl (M.v.d.S.); pacher@mail.nih.gov (P.P.) and liuzhj@shanghaitech.edu.cn (Z.-J.L.).

SUPPLEMENTAL FIGURES AND TABLES

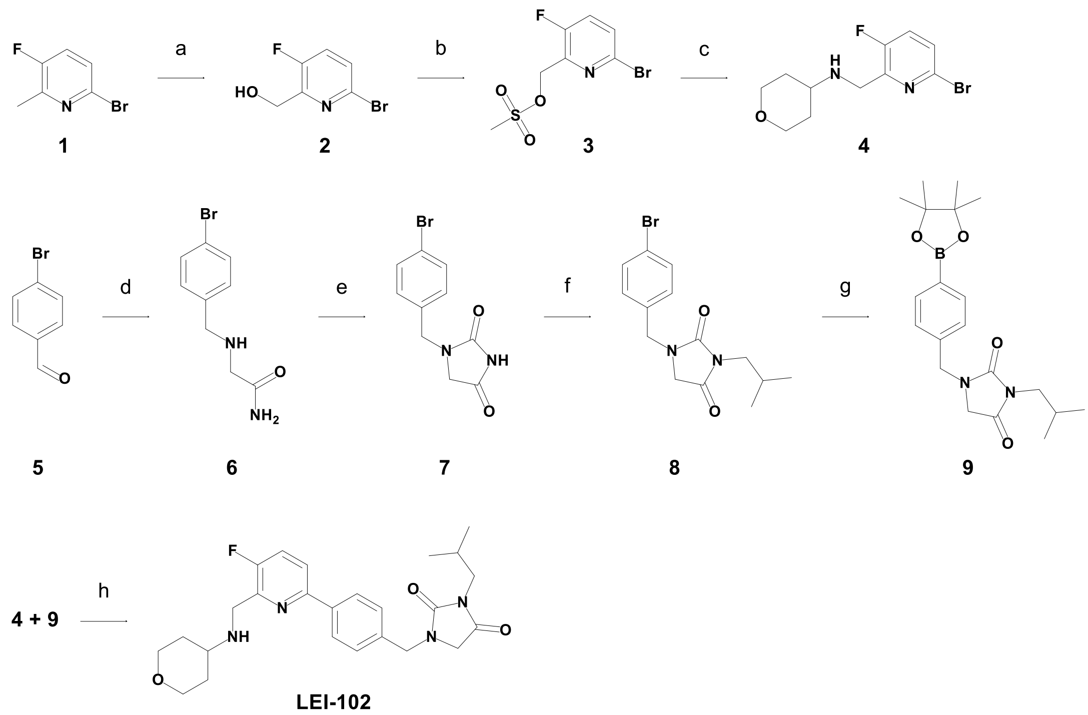

Supplementary Fig. 1 | Synthetic route of LEI-102. Reagents and conditions: a) step 1: *m*-CPBA (1.8 eq), 0 °C-rt, DCM, 4 days; step 2: TFAA (2.2 eq), 55 °C, 3 h; step 3: K_2CO_3 (2.4 eq), THF:MeOH (20:1), 17 h, 35% (three steps); b) Et_3N (2.3 eq), MsCl (1.7 eq), THF, 0 °C-rt, 1 h, 75%; c) K_2CO_3 (2.2 eq), tetrahydro-2H-pyran-4-amine (1.3 eq), ACN, 50 °C, 3 h, 67%; d) step 1: 2-aminoacetamide hydrochloride (1.0 eq), NaOH (1.1 eq), MeOH:H_2O (5:1), rt, 18 h; $NaBH_4$ (2.1 eq), 18 h, 91% (two steps); e) CDI (2.1 eq), DMAP (2.1 eq), ACN, 60 °C, 70 h, 37%; f) K_2CO_3 (3.0 eq), 1-bromo-2-methylpropane (2.0 eq), DMF, rt, 20 h, 88%; g) KOAc (4.4 eq), bis(pinacolato)diboron (1.5 eq), Pd(dppf)Cl_2 (0.06 eq), DMF, 75 °C, 20 h; h) **4** (1.0 eq), **9** (1.5 eq), K_2CO_3 (6.0 eq), Pd(PPh_3)$_4$ (0.1 eq), toluene:EtOH (4:1), 75 °C, 18 h, 45% (two steps).

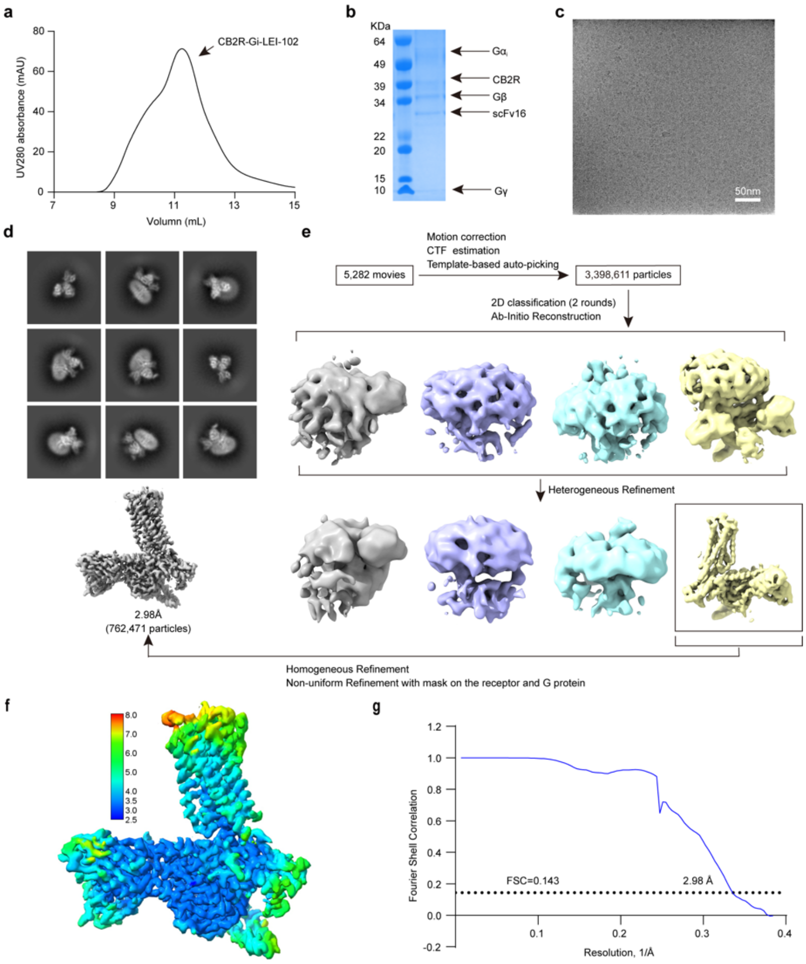

Supplementary Fig. 2 | CB$_2$R-G$_i$-scfv16-LEI-102 complex preparation and cryo-EM data processing. **(a)** Representative size-exclusion chromatography elution profile of CB$_2$R complex. Fractions corresponding to the main peak of monomers are indicated. **(b)** Coomassie blue staining of the CB$_2$R-G$_i$-scFv16 complex. **(c)** Representative cryo-EM micrograph of the CB$_2$R-G$_i$-scFv16 complex. Scale bar: 50 nm. **(d)** Representative 2D averages (box size: 256 Å) showing diverse secondary structure features. **(e)** Flow chart of cryo-EM single particle analysis of the CB$_2$R-G$_i$-scFv16 complex. **(f)** Local resolution map of the CB$_2$R-G$_i$-scFv16 complex. **(g)** Fourier shell correlation curves of the CB$_2$R-G$_i$-scFv16 complex.

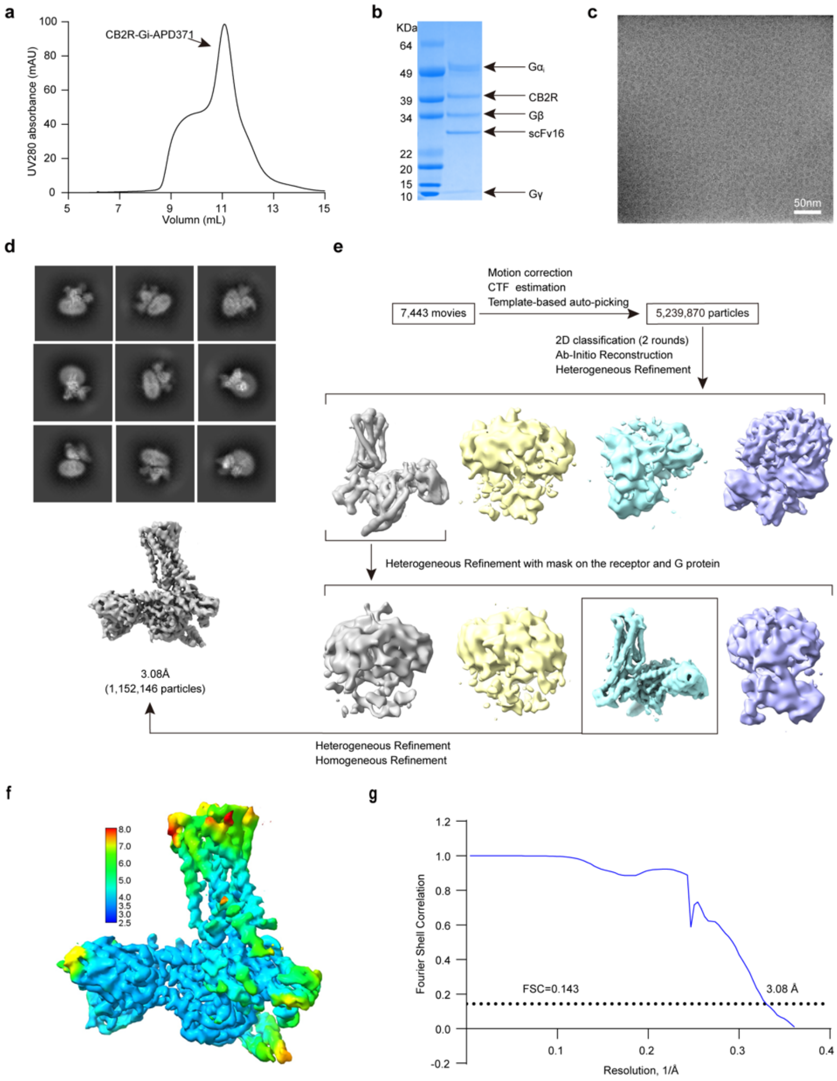

Supplementary Fig. 3 | CB$_2$R-G$_i$-scFv16-APD371 complex preparation and cryo-EM data processing. **(a)** Representative size-exclusion chromatography elution profile of CB$_2$R complex. Fractions corresponding to the main peak of monomers are indicated. **(b)** Coomassie blue staining of the CB$_2$R-G$_i$-scFv16 complex. **(c)** Representative cryo-EM micrograph of the CB$_2$R-G$_i$-scFv16 complex. Scale bar: 50 nm. **(d)** Representative 2D averages (box size: 256 Å) showing diverse secondary structure features. **(e)** Flow chart of cryo-EM single particle analysis of the CB$_2$R-G$_i$-scFv16 complex. **(f)** Local resolution map of the CB$_2$R-G$_i$-scFv16 complex. **(g)** Fourier shell correlation curves of the CB$_2$R-G$_i$-scFv16 complex.

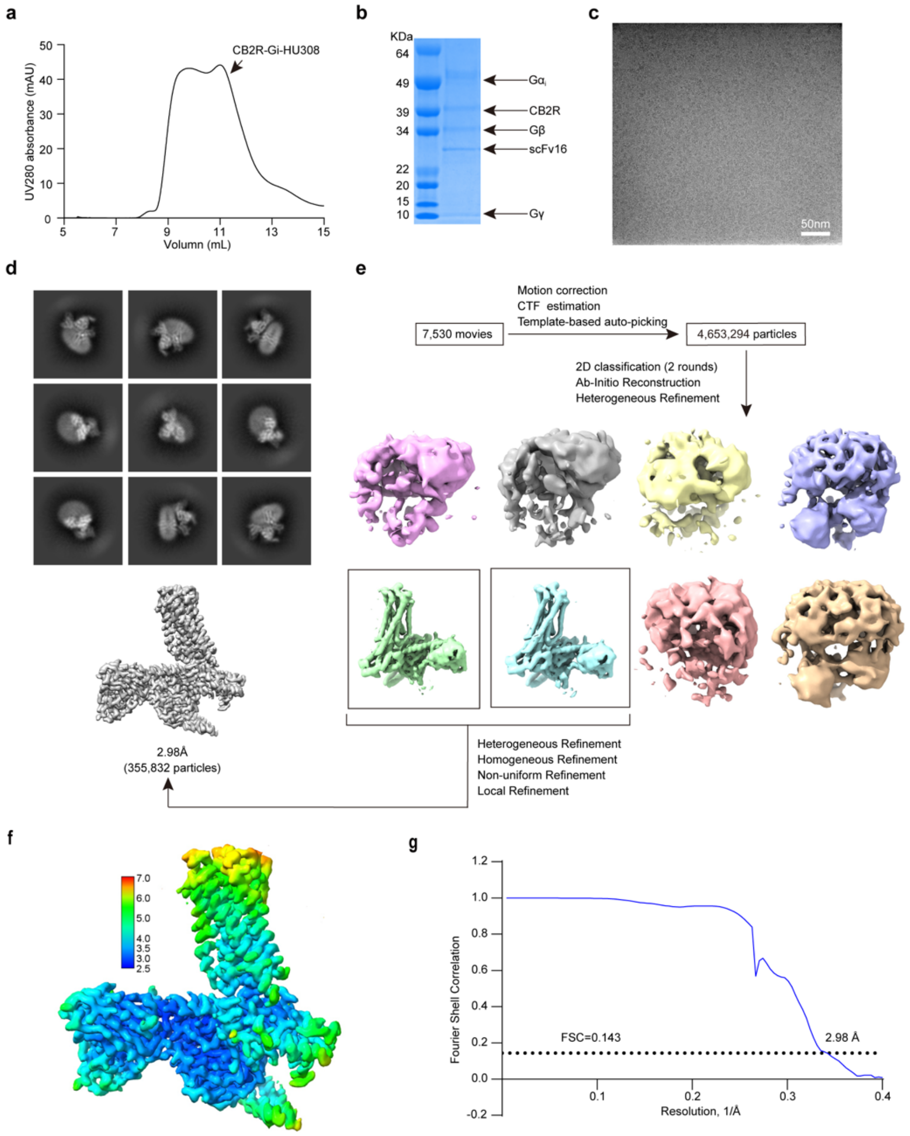

Supplementary Fig. 4 | CB$_2$R-G$_i$-scFv16-HU308 complex preparation and cryo-EM data processing. **(a)** Representative size-exclusion chromatography elution profile of CB$_2$R complex. Fractions corresponding to the main peak of monomers are indicated. **(b)** Coomassie blue staining of the CB$_2$R-G$_i$-scFv16 complex. **(c)** Representative cryo-EM micrograph of the CB$_2$R-G$_i$-scFv16 complex. Scale bar: 50 nm. **(d)** Representative 2D averages (box size: 256 Å) showing diverse secondary structure features. **(e)** Flow chart of cryo-EM single particle analysis of the CB$_2$R-G$_i$-scFv16 complex. **(f)** Local resolution map of the CB$_2$R-G$_i$-scFv16 complex. **(g)** Fourier shell correlation curves of the CB$_2$R-G$_i$-scFv16 complex.

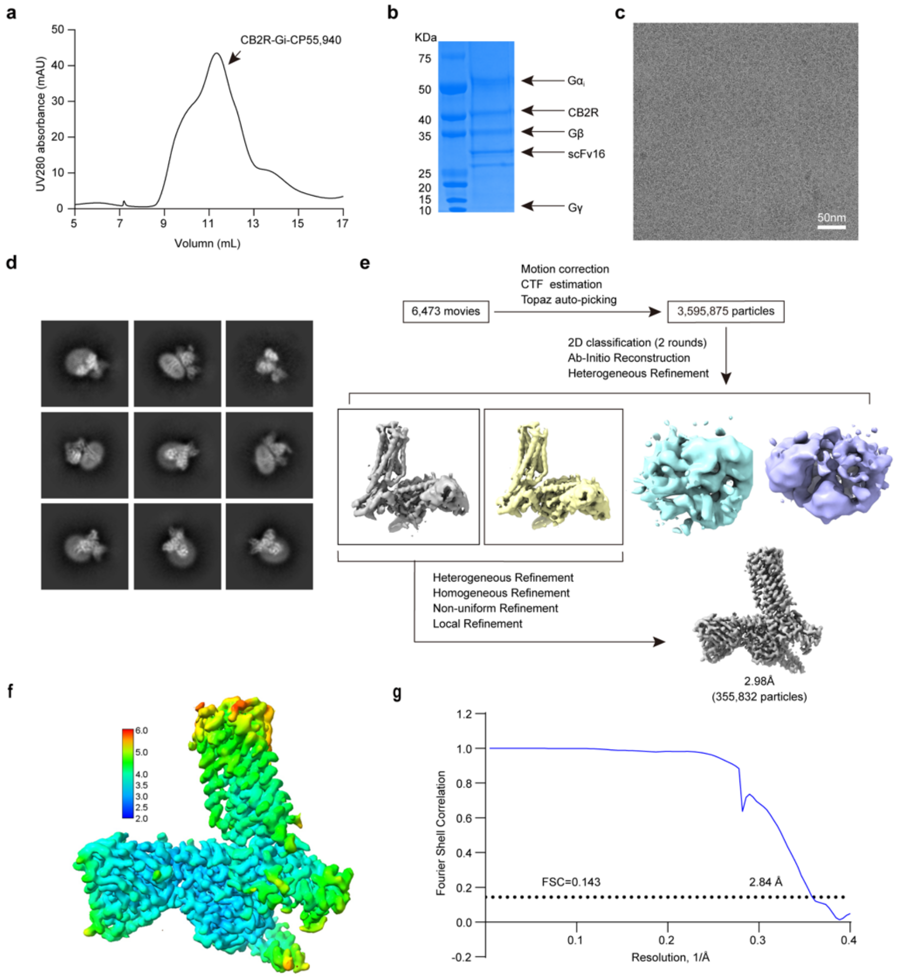

Supplementary Fig. 5 | CB_2R-G_i-scFv16-CP55,940 complex preparation and cryo-EM data processing. (a) Representative size-exclusion chromatography elution profile of CB_2R complex. Fractions corresponding to the main peak of monomers are indicated. **(b)** Coomassie blue staining of the CB_2R-G_i-scFv16 complex. **(c)** Representative cryo-EM micrograph of the CB_2R-G_i-scFv16 complex. Scale bar: 50 nm. **(d)** Representative 2D averages (box size: 256 Å) showing diverse secondary structure features. **(e)** Flow chart of cryo-EM single particle analysis of the CB_2R-G_i-scFv16 complex. **(f)** Local resolution map of the CB_2R-G_i-scFv16 complex. **(g)** Fourier shell correlation curves of the CB_2R-G_i-scFv16 complex.

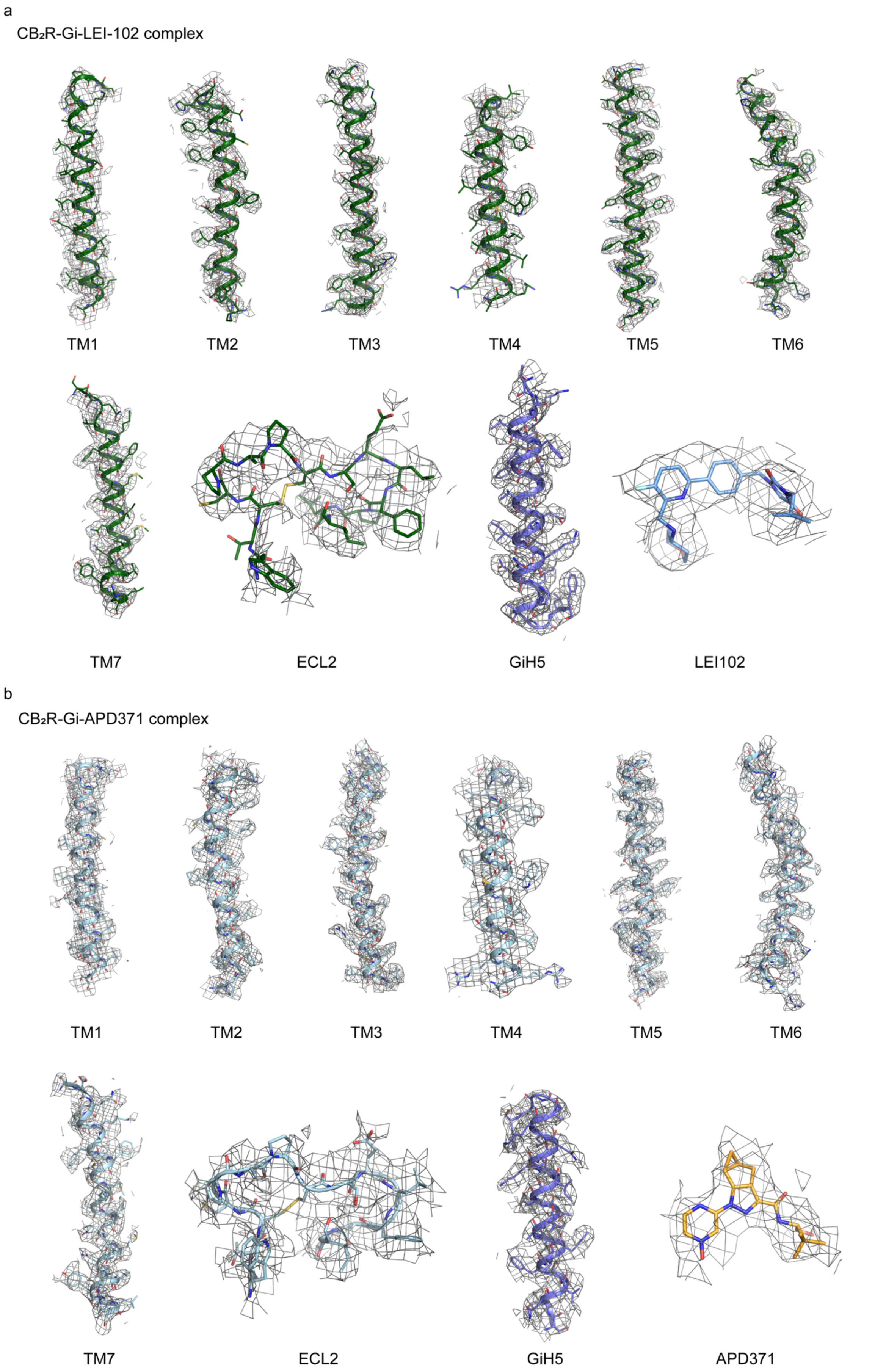
a
CB₂R-Gi-LEI-102 complex
TM1
TM2
TM3
TM4
TM5
TM6
TM7
ECL2
GiH5
LEI102
b
CB₂R-Gi-APD371 complex
TM1
TM2
TM3
TM4
TM5
TM6
TM7
ECL2
GiH5
APD371

c

CB$_2$R-Gi-HU308 complex

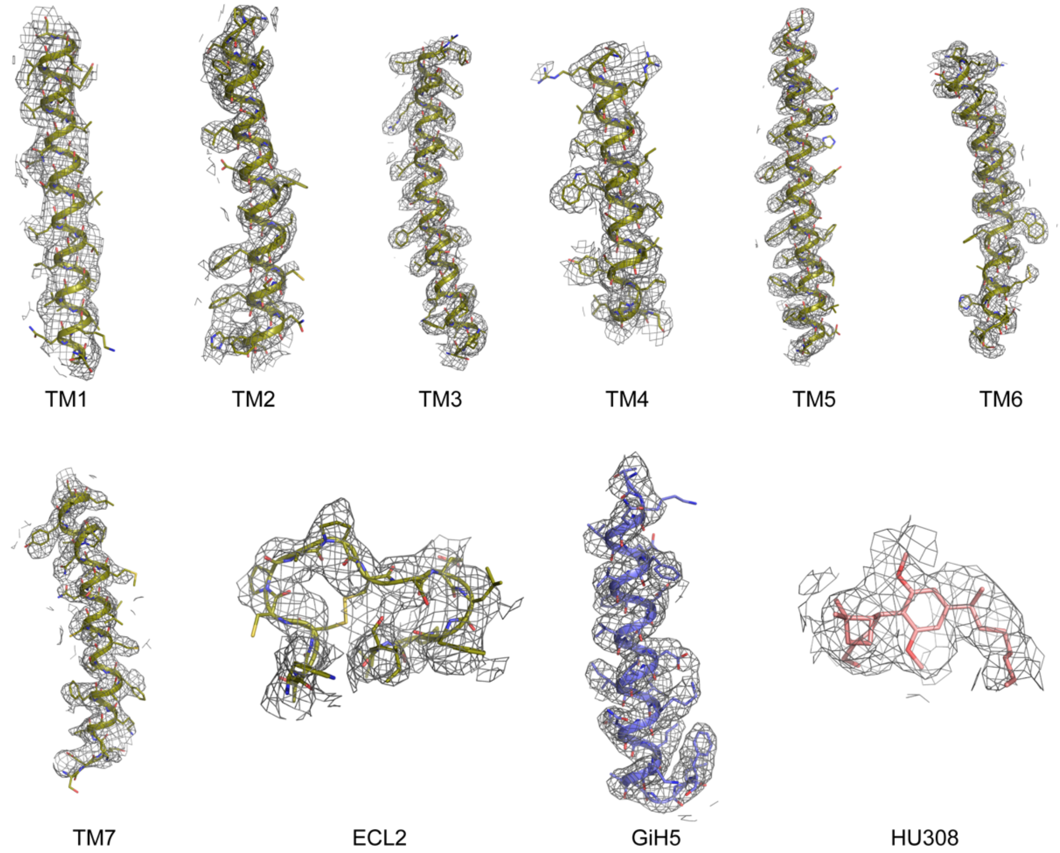

d

CB$_2$R-Gi-CP55,940 complex

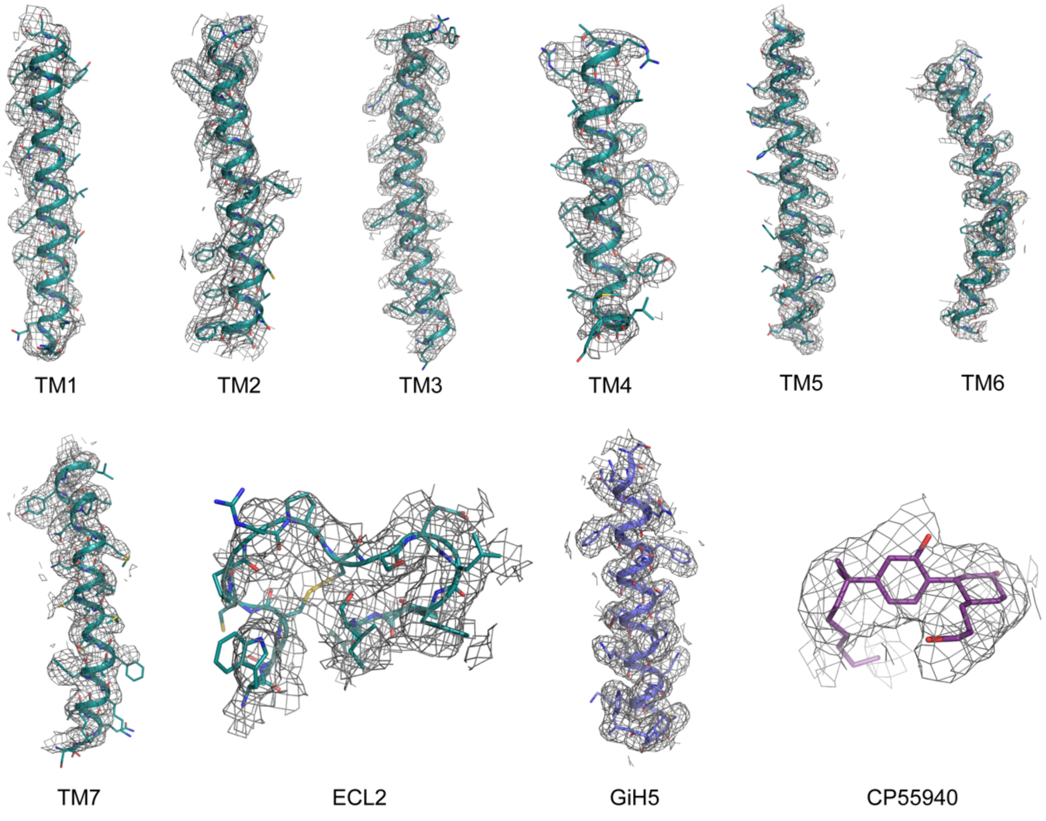

Supplementary Fig. 6 | Representative cryo-EM density map of the CB_2R-G_i-scFv16 complex. (a-d) EM density map and models are shown for all transmembrane helices, ECL2, ligand and α5 helix of $G\alpha_i$ ($G\alpha_iH5$) in **(a)** LEI-102, **(b)** APD371, **(c)** HU308 and **(d)** CP55,940 bound CB_2R-G_i-scFv16 complex.

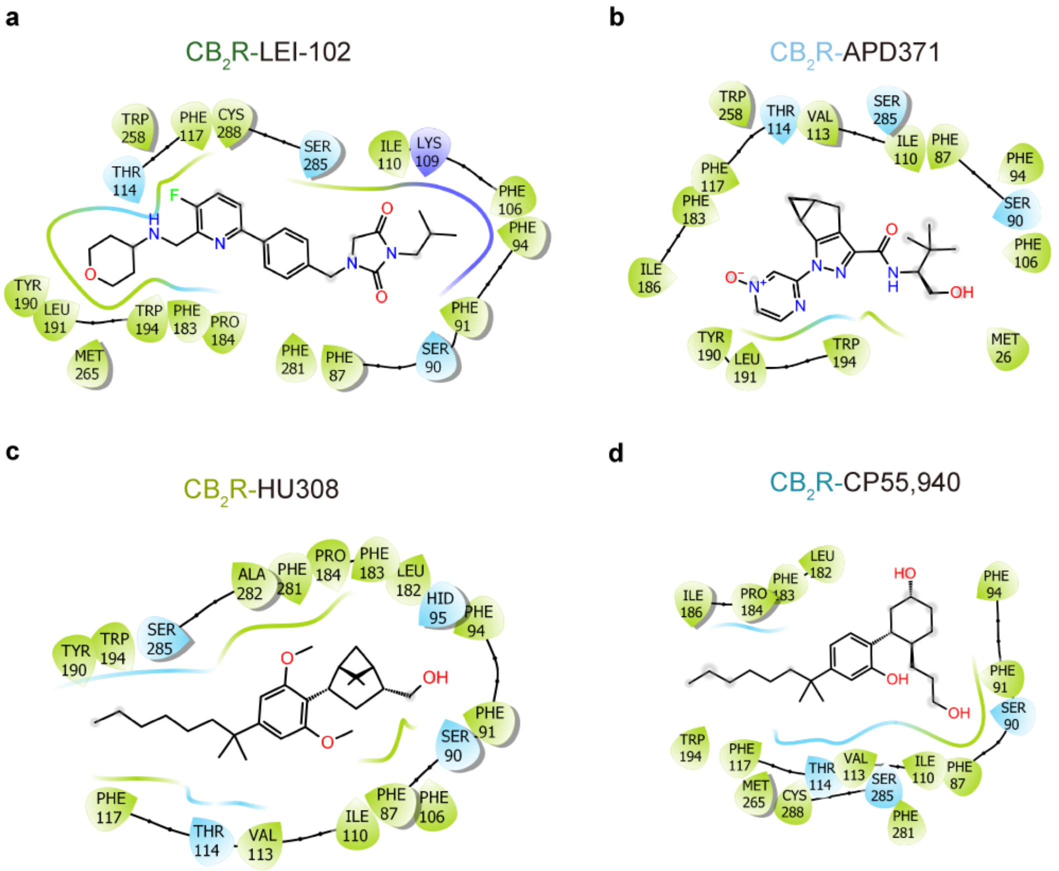

Supplementary Fig. 7 | Schematic representation of interactions between CB₂R and Agonists. (a-d) Schematic 2D representation of the binding pocket of agonist **(a)** LEI-102, **(b)** APD371, **(c)** HU308 and **(d)** CP55,940. Hydrophobic amino acids are colored in green, polar amino acids are colored in cyan, basic amino acids are colored in purple.

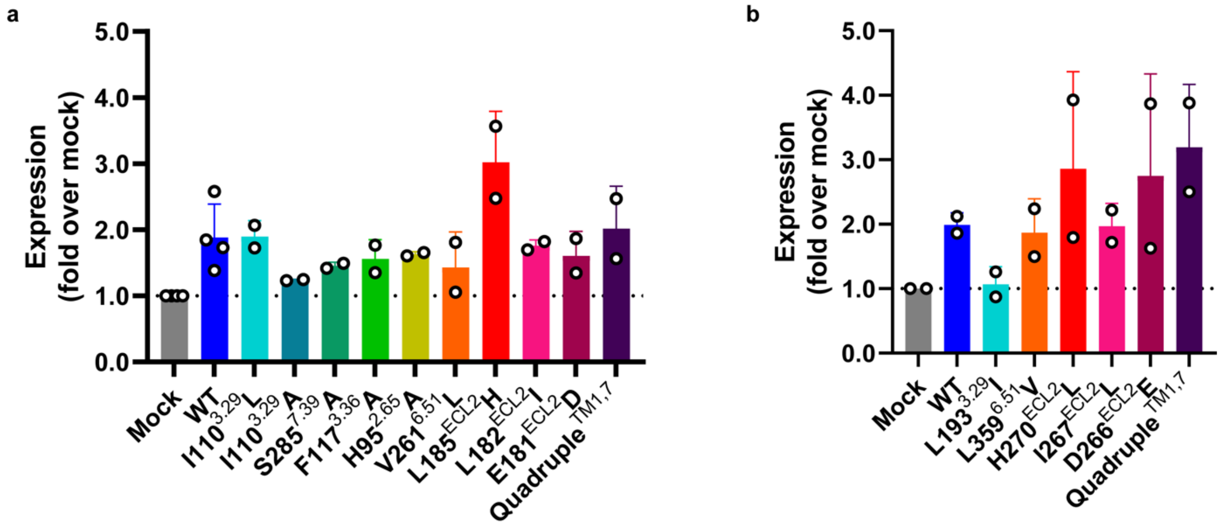

Supplementary Fig. 8 | Cell surface receptor expression

Receptor expression as determined by ELISA for **(a)** CB_2R and **(b)** CB_1R wild type (WT) and mutants. Data are expressed as mean ± SD of at least two experiments performed in quintuplicate (specific *n* values are given in Supplementary Table 5). Source data are provided as a Source Data file.

Supplementary Table 1. Physico-chemical properties of the investigated ligands. All values calculated using RDKit KNIME nodes version 4.5.0.v202207051536, apart from CLogP which was calculated using ChemDraw 19.0.

**Compound**	**CLogP**	**SLogP**	**TPSA (Å)**	**MW (g/mol)**	**NumRotatableBonds**	**NumHBD**	**NumHBA**
Δ^9-THC	7.24	5.74	29.5	314.5	4	1	2
2-AG	6.89	5.03	66.8	378.6	17	2	4
AEA	6.18	5.24	49.3	347.5	16	2	2
CP55,940	5.82	5.66	60.7	376.6	10	3	3
HU308	8.00	6.63	38.7	414.6	10	1	3
APD371	-0.35	0.70	107.0	357.4	4	2	6
LEI-102	2.07	3.58	74.8	454.5	8	1	5

Supplementary Table 2. Functional activity, affinity and kinetic parameters of synthetic agonists and endocannabinoids on hCB_2R

Compound	pEC_{50}[a]	E_{max} (%)[b]	*n*	pK_i (K_i, nM)[c]	*n*	k_{on} ($M^{-1}s^{-1}$)[d]	ET (s)[e]	k_{off} (s^{-1})[f]	RT (min)[g]	K_D (nM)[h]	*n*
**LEI-102**	6.9 ± 0.2	76 ± 1	3	8.0 ± 0.1 (9.7)	3	$(6.3 \pm 1.1) \times 10^4$	16.0 ± 2.82	$(1.0 \pm 0.2) \times 10^{-3}$	16 ± 3.3	16.5	3
**APD371**	7.9 ± 0.1	134 ± 12	3	7.5 ± 0.1 (35.3)	3	$(2.5 \pm 0.4) \times 10^4$	40.1 ± 5.90	$(3.7 \pm 0.5) \times 10^{-4}$	45 ± 6.6	14.9	3
**HU308**	7.1 ± 0.2	91 ± 8	3	7.0 ± 0.1 (92.4)	3	$(7.0 \pm 2.3) \times 10^3$	143 ± 47.9	$(2.3 \pm 0.4) \times 10^{-4}$	71 ± 11	33.7	3
**CP55,940**	8.5 ± 0.3	98 ± 4	6	8.9 ± 0.1 (1.4)	3	$(1.8 \pm 0.4) \times 10^6$	0.57 ± 0.13	$(5.2 \pm 0.9) \times 10^{-4}$	32 ± 5.5	0.3	3
**AEA**	6.3 ± 0.2	60 ± 5	3	6.3 ± 0.1 (484.5)	3	$(6.6 \pm 0.5) \times 10^3$	152 ± 10.4	$(2.4 \pm 0.1) \times 10^{-3}$	6.8 ± 0.4	371.7	3
**2-AG**	5.9 ± 0.1	93 ± 22	3	7.0 ± 0.1 (97.3)	3	$(5.3 \pm 1.0) \times 10^4$	18.8 ± 3.61	$(2.3 \pm 0.3) \times 10^{-3}$	7.4 ± 1.0	42.7	3

[a, b] Potency (pEC_{50}) and efficacy (E_{max}) values were obtained from [^{35}S]GTPγS assays on HEK293T membranes transiently expressing CB_2R WT. The percentage maximum effect (E_{max} in %) was calculated compared to CP55,940. [c, d, f] Binding affinities (pK_i), association (k_{on}) and dissociation (k_{off}) rate constants were determined in [^{3}H]RO6957022 binding assays on CHOK1_hCB$_2$bgal membranes at 10 °C. [e] Engagement time (ET) of the compound to the receptor at 1 µM agonist was determined by $ET = 1/(k_{on} \cdot 1 \times 10^{-6})$ and is expressed in seconds (s), whereas k_{on} is expressed in $M^{-1}s^{-1}$. [g] Residence time (RT) was determined by $RT = 1/(60 \cdot k_{off})$ and is expressed in min, whereas k_{off} is expressed in s^{-1}. [h] Kinetic K_D values, defined by $K_D = k_{off}/k_{on}$. Values represent the mean ± SEM of at least three independent experiments performed in duplicate (*n* indicates the number of biological replicates). Source data are provided as a Source Data file.

Supplementary Table 3. Characterization of cannabinoid CB_1 receptor mutations in $[^3H]$CP55,940 displacement assays

			LEI-102	APD371	HU308	CP55,940	AEA	2-AG	
		pK_D (K_D, nM)	Displacement (%)[a]			pK_i[b]			*n*
	**WT**	8.3 ± 0.1 (5.24)	17 ± 7	-19 ± 17	6 ± 7	8.3 ± 0.1	6.3 ± 0.1	5.6 ± 0.2	3
**Binding pocket**	**$L193^{3.29}I$**	n.d.	n.d.	n.d.	n.d.	n.d.	n.d.	n.d.	3
	**$L359^{6.51}V$**	8.6 ± 0.2 (2.80)	25 ± 6 [0.8796]	18 ± 9 [0.4248]	41 ± 10 [0.6809]	8.5 ± 0.1 [0.3912]	6.3 ± 0.1 [>0.9999]	5.9 ± 0.1 [0.4246]	3
**Ligand entry**	**$H270^{ECL2}L$**	8.4 ± 0.2 (3.70)	47 ± 5 [0.0839]	8 ± 3 [0.6025]	46 ± 3 [0.8951]	8.4 ± 0.1 [0.7882]	6.3 ± 0.0 [0.9992]	5.5 ± 0.2 [0.9992]	3
	**$I267^{ECL2}L$**	8.6 ± 0.1 (2.57)	25 ± 3 [0.7745]	-24 ± 17 [0.9998]	-7 ± 19 [0.2577]	8.6 ± 0.1 [0.0802]	6.4 ± 0.1 [0.9895]	6.1 ± 0.2 [0.3849]	3
	**$D266^{ECL2}E$**	8.7 ± 0.2 (1.91)	38 ± 10 [0.4418]	12 ± 7 [0.5227]	34 ± 5 [0.3445]	8.7 ± 0.1 [0.1266]	6.7 ± 0.1 [0.3000]	5.9 ± 0.1 [0.4302]	3
	**$Quadruple^{TM1,7}$**	8.1 ± 0.2 (8.36)	26 ± 10 [0.9110]	0 ± 25 [0.9689]	-13 ± 22 [0.8718]	8.1 ± 0.2 [0.7771]	6.4 ± 0.0 [0.9796]	5.3 ± 0.0 [0.6300]	3

Mutations are shown in the numbering of the cannabinoid CB_1 receptor (CB_1R) amino acid sequence, as well as the Ballesteros and Weinstein GPCR numbering system. [a,b] Percentage of $[^3H]$CP55,940 displacement by 10 µM compound or binding affinity (pK_i) were determined in $[^3H]$CP55,940 displacement assays on HEK293T membranes transiently expressing CB_1R constructs. All values are presented as the mean ± SEM of at least three independent experiments performed in duplicate (*n* indicates the number of biological replicates). One-way Welch ANOVA with Dunnett's T3 posthoc test was used to analyze differences in pK_D or pK_i values compared to WT. Exact p values are given between square brackets. n.d. is not determined. Source data are provided as a Source Data file.

Supplementary Table 4. Cryo-EM data collection, model refinement, and validation statistics.

	CB_2R-HU308-G_i	CB_2R-LEI-102-G_i	CB_2R-APD371-G_i	CB_2R-CP55,940-G_i
**Data collection and processing**				
Magnification	105,000	105,000	105,000	105,000
Voltage (kV)	300	300	300	300
Electron exposure (e–/Å^2)	60	60	60	60
Defocus range (μm)	-1.0~-2.0	-1.0~-2.0	-1.0~-2.0	-1.0~-2.0
Pixel size (Å)	0.52	0.52	0.52	0.52
Symmetry imposed	C1	C1	C1	C1
Images（no.）	7,530	5,282	7,443	6,473
Initial particle images (no.)	4,653,294	3,398,611	5,239,870	3,595,875
Final particle images (no.)	355,832	762,471	1,152,146	440,292
Map resolution (Å)	2.97	2.98	3.08	2.84
FSC threshold	0.143	0.143	0.143	0.143
Map resolution range (Å)				
	2.9~6.0	2.9~6.0	2.9~6.0	2.7~5.0
**Refinement**				
Initial model used (PDB code)	6KPF	6KPF	6KPF	6KPF
Map sharpening *B* factor (Å^2)	110.8	-104.2	-118.6	-100.9
Model composition				
Non-hydrogen atoms	8,817	8,812	8,801	8,888
Protein residues	1,137	1,137	1,137	1,139
Ligands	1	1	1	1
B factors (Å^2)				
Protein	68.91	68.00	61.04	68.22
Ligand	67.91	76.90	104.87	85.48
R.m.s. deviations				
Bond lengths (Å)	0006	0.003	0.009	0.010
Bond angles (°)	0.69	0.57	1.24	1.80
Validation				
MolProbity score	1.83	1.82	1.64	1.46
Clashscore	12.11	12.29	5.04	7.74
Poor rotamers (%)	0.00	0.00	0.32	0.00
Ramachandran plot				
Favored (%)	96.43	96.60	94.46	97.86
Allowed (%)	3.57	3.40	5.54	2.14
Disallowed (%)	0.00	0.00	0.00	0.00

Supplementary Table 5. Cell surface expression levels of cannabinoid receptor constructs in ELISA.

	CB_2R	Expression (fold over mock)	*n*	CB_1R	Expression (fold over mock)	*n*
	**WT**	1.9 ± 0.5	4	**WT**	2.0 ± 0.2	2
**Binding pocket**	**$I110^{3.29}L$**	1.9 ± 0.2	2	**$L193^{3.29}I$**	1.1 ± 0.3	2
	**$I110^{3.29}A$**	1.2 ± 0.0	2	**$L193^{3.29}A$**	n.d	2
	**$S285^{7.39}A$**	1.5 ± 0.1	2	**$S383^{7.39}A$**	n.d	2
	**$F117^{3.36}A$**	1.6 ± 0.3	2	**$F200^{3.36}A$**	n.d	2
	**$H95^{2.65}A$**	1.6 ± 0.0	2	**$H178^{2.65}A$**	n.d	2
	**$V261^{6.51}L$**	1.4 ± 0.5	2	**$L359^{6.51}V$**	1.9 ± 0.5	2
**Ligand entry**	**$L185^{ECL2}H$**	3.0 ± 0.8	2	**$H270^{ECL2}L$**	2.9 ± 1.5	2
	**$L182^{ECL2}I$**	1.8 ± 0.1	2	**$I267^{ECL2}L$**	2.0 ± 0.4	2
	**$E181^{ECL2}D$**	1.6 ± 0.4	2	**$D266^{ECL2}E$**	2.7 ± 1.6	2
	**$Quadruple^{TM1,7}$**	2.0 ± 0.6	2	**$Quadruple^{TM1,7}$**	3.2 ± 1.0	2

Mutations are shown in the numbering of the cannabinoid CB_2 (CB_2R) or CB_1 receptor (CB_1R) amino acid sequence as well as the Ballesteros and Weinstein GPCR numbering system. Data are presented as fold over mock (empty pcDNA3.1 vector) and are mean ± SD of at least two individual experiments performed in quintuplicate (*n* indicates the number of biological replicates). n.d. is not determined. Source data are provided as a Source Data file.

Supplementary Table 6. Characterization of cannabinoid CB_2 receptor mutations in G protein activation assays.

				**LEI-102**			**APD371**			**HU308**			**CP55,940**			**AEA**			**2-AG**		
	**Construct**	**Basal**[a]	***n***	**pEC$_{50}$**[b]	**E$_{max}$ (%)**[c]	***n***	**pEC$_{50}$**	**E$_{max}$ (%)**	***n***	**pEC$_{50}$**	**E$_{max}$ (%)**	***n***	**pEC$_{50}$**	**E$_{max}$ (%)**	***n***	**pEC$_{50}$**	**E$_{max}$ (%)**	***n***	**pEC$_{50}$**	**E$_{max}$ (%)**	***n***
	**WT**	1.0 ± 0.0	9	6.9 ± 0.2	103 ± 3	10	7.9 ± 0.1	109 ± 3	10	7.1 ± 0.2	105 ± 3	10	8.5 ± 0.3	99 ± 3	9	6.3 ± 0.2	106 ± 7	3	5.9 ± 0.1	85 ± 2	3
**Binding pocket**	**I110$^{3.29}$L**	1.0 ± 0.0 [0.5080]	3	7.8 ± 0.1** [0.0056]	91 ± 3 [0.3262]	3	6.7 ± 0.3 [0.1190]	101 ± 7 [0.9253]	3	6.7 ± 0.4 [0.8664]	122 ± 12 [0.8030]	3	9.4 ± 0.2 [0.0625]	96 ± 3 [0.9938]	3	n.d.	n.d.	3	n.d.	n.d.	3
	**I110$^{3.29}$A**	1.0 ± 0.1 [>0.9999]	3	7.0 ± 0.6 [>0.9999]	36 ± 13 [0.1391]	3	6.6 ± 0.6 [0.4641]	28 ± 9** [0.0052]	4	6.4 ± 0.5 [0.7656]	27 ± 10** [0.0094]	4	8.7 ± 0.4 [0.9975]	30 ± 2**** [<0.0001]	3	n.d.	n.d.	3	n.d.	n.d.	3
	**S285$^{7.39}$A**	0.9 ± 0.0 [0.1553]	3	6.3 ± 0.3 [0.5159]	45 ± 8* [0.0358]	3	6.5 ± 0.3 [0.1034]	49 ± 6* [0.0139]	3	5.9 ± 0.1*** [0.0002]	65 ± 18 [0.5179]	3	6.7 ± 0.1** [0.0010]	25 ± 5** [0.0020]	3	n.d.	n.d.	3	n.d.	n.d.	3
	**F117$^{3.36}$A**	0.8 ± 0.1 [0.4725]	3	n.d.	11 ± 11 [0.0578]	3	n.d.	2 ± 6** [0.0028]	3	n.d.	5 ± 11* [0.0475]	3	n.d.	-1 ± 14 [0.0755]	3	n.d.	n.d.	3	n.d.	n.d.	3
	**H95$^{2.65}$A**	0.9 ± 0.0 [0.3113]	3	<5	64 ± 7 [0.0630]	3	5.7 ± 0.2** [0.0043]	50 ± 1**** [<0.0001]	3	6.6 ± 0.6 [0.9210]	67 ± 16 [0.4831]	3	6.9 ± 0.2** [0.0029]	68 ± 11 [0.3525]	3	n.d.	n.d.	3	n.d.	n.d.	3
	**V261$^{6.51}$L**	0.8 ± 0.0 [0.1710]	3	6.4 ± 0.0 [0.6680]	8 ± 4**** [<0.0001]	3	6.9 ± 0.2* [0.0364]	6 ± 2**** [<0.0001]	3	<5	26 ± 7** [0.0086]	3	<5	12 ± 7** [0.0085]	3	n.d.	n.d.	3	n.d.	n.d.	3
**Ligand entry**	**L185^{ECL2}H**	1.2 ± 0.1 [0.2049]	6	6.9 ± 0.1 [>0.9999]	217 ± 21 [0.1210]	3	8.0 ± 0.1 [0.9490]	183 ± 16 [0.1714]	3	6.8 ± 0.2 [0.6863]	146 ± 8 [0.0741]	3	8.9 ± 0.4 [0.9552]	142 ± 16 [0.3320]	4	6.6 ± 0.0 [0.2269]	210 ± 61 [0.5043]	3	5.9 ± 0.2 [0.9835]	171 ± 16 [0.0852]	3
	**L182^{ECL2}I**	0.8 ± 0.0* [0.0180]	6	7.4 ± 0.8 [0.9926]	11 ± 8* [0.0106]	3	6.6 ± 0.4 [0.3556]	17 ± 4*** [0.0002]	3	<5	10 ± 7** [0.0059]	3	8.8 ± 0.8 [0.9995]	19 ± 2**** [<0.0001]	3	<5	5 ± 17* [0.0329]	3	<5	15 ± 9* [0.0433]	3
	**E181^{ECL2}D**	0.9 ± 0.0 [0.6017]	6	6.9 ± 0.6 [>0.9999]	28 ± 13 [0.1146]	3	6.9 ± 0.6 [0.6868]	39 ± 6** [0.0096]	3	7.0 ± 0.7 [0.9998]	32 ± 3**** [<0.0001]	3	7.9 ± 0.5 [0.8972]	19 ± 10 [0.0669]	3	<5	14 ± 5** [0.0012]	3	<5	22 ± 15 [0.1357]	3

	**Quadruple™** [1,7]	0.9 ± 0.0<br>[0.2061]	3	6.2 ± 0.2<br>[0.2672]	27 ± 5**<br>[0.0012]	3	8.4 ± 0.1<br>[0.2470]	35 ± 7*<br>[0.0117]	3	n.d.#	1 ± 9*<br>[0.0323]	3	8.1 ± 0.2<br>[0.8571]	18 ± 14<br>[0.1110]	3	<5	24 ± 7**<br>[0.0036]	3	<5	6 ± 4***<br>[0.0010]	3

Mutations are shown in the numbering of the cannabinoid CB_2 receptor (CB_2R) amino acid sequence as well as the Ballesteros and Weinstein GPCR numbering system. [a,b,c] Basal activity, potency (pEC_{50}) and efficacy (E_{max}) values were obtained from [^{35}S]GTPγS assays on HEK293T membranes transiently expressing CB_2R constructs. The percentage maximum effect at 10 μM (E_{max} in %) and the fold basal activity values were calculated compared to WT. pEC_{50} <5 was reported when the curve fit was not finished. Binding pocket mutations were not assessed for endocannabinoids. Values are presented as the mean ± SEM of at least three independent experiments performed in duplicate (*n* indicates the number of biological replicates). One-way Welch ANOVA with Dunnett's T3 posthoc test or Welch's t-test was used to analyze differences in pEC_{50} and E_{max} values compared to WT (*p < 0.05, ** p < 0.01, *** p < 0.001, **** p < 0.00001). Exact p values are given between square brackets. n.d. is not determined. Source data are provided as a Source Data file.

Supplementary Table 7. Characterization of cannabinoid CB$_2$ receptor mutations in [^{3}H]CP55,940 displacement assays.

				**LEI-102**		**APD371**		**HU308**		**CP55,940**		**AEA**		**2-AG**	
		**pK$_D$ (K$_D$, nM)**[a]	*n*	**pK$_i$**[b]	*n*	**pK$_i$**[b]	*n*	**pK$_i$**[b]	*n*	**pK$_i$**[b]	*n*	**pK$_i$**[b]	*n*	**pK$_i$**[b]	*n*
	**WT**	9.1 ± 0.0 (0.77)	3	7.5 ± 0.1	3	8.0 ± 0.0	3	7.8 ± 0.2	3	9.2 ± 0.2	3	6.2 ± 0.1	3	5.8 ± 0.1	3
Binding pocket	**I110$^{3.29}$L**	9.2 ± 0.2 (0.66)	4	7.9 ± 0.0 [0.0674]	3	7.1 ± 0.0*** [0.0008]	3	7.3 ± 0.1 [0.2954]	3	9.1 ± 0.1 [0.9959]	3	6.1 ± 0.1 [0.7643]	3	5.8 ± 0.1 [0.9226]	3
Ligand entry	**L185^{ECL2}H**	9.2 ± 0.1 (0.60)	3	7.3 ± 0.1 [0.6573]	3	7.7 ± 0.1 [0.0856]	3	7.2 ± 0.0 [0.2561]	3	9.4 ± 0.1 [0.6321]	3	6.3 ± 0.1 [0.6860]	3	6.4 ± 0.2 [0.1339]	3
	**Quadruple^{TM1,7}**	9.0 ± 0.1 (1.08)	3	6.8 ± 0.2 [0.1776]	3	6.7 ± 0.3 [0.0916]	3	7.1 ± 0.4 [0.3843]	3	9.2 ± 0.1 [0.9927]	3	5.6 ± 0.3 [0.3218]	3	<5	3

Mutations are shown in the numbering of the cannabinoid CB$_2$ receptor (CB$_2$R) amino acid sequence as well as the Ballesteros and Weinstein GPCR numbering system. [a,b] Binding affinities (pK$_D$, pK$_i$) were determined in [^{3}H]CP55,940 displacement assays on HEK293T membranes transiently expressing CB$_2$R constructs. pK$_i$ <5 was reported when the curve fit was not finished. All values are presented as the mean ± SEM of at least three independent experiments performed in duplicate (*n* indicates the number of biological replicates). One-way Welch ANOVA with Dunnett's T3 posthoc test was used to analyze differences in pK$_i$ values compared to WT (*$p < 0.05$, ** $p < 0.01$, *** $p < 0.001$). Exact p values are given between square brackets. Mutations I110$^{3.29}$L, S285$^{7.39}$A, F117$^{3.36}$A, H95$^{2.65}$A, V261$^{6.51}$L, L185^{ECL2}I and E181^{ECL2}D could not be assessed due to lack of specific binding window. Source data are provided as a Source Data file.

Supplementary Table 8. Characterization of cannabinoid CB_1 receptor mutations in G protein activation assays

	Construct	Basal[a]	LEI-102	APD371	HU308	CP55,940	AEA	2-AG	*n*
	**WT**	1.0 ± 0.0	1.0 ± 0.1	1.0 ± 0.0	0.8 ± 0.0	1.2 ± 0.1	1.2 ± 0.1	1.3 ± 0.1	3
Binding pocket	**L193$^{3.29}$I**	n.d.	n.d.	n.d.	n.d.	n.d.	n.d.	n.d.	n.d.
	**L359$^{6.51}$V**	0.7 ± 0.0* [0.0218]	1.1 ± 0.1 [0.8066]	1.1 ± 0.1 [0.5608]	0.9 ± 0.1 [0.6307]	1.4 ± 0.2 [0.9142]	1.4 ± 0.2 [0.8892]	1.4 ± 0.2 [0.9084]	3
Ligand entry	**H270^{ECL2}L**	0.7 ± 0.0** [0.0043]	1.2 ± 0.0 [0.3066]	1.1 ± 0.0 [0.2564]	1.2 ± 0.1* [0.0273]	1.6 ± 0.1 [0.1137]	1.4 ± 0.1 [0.5125]	1.5 ± 0.1 [0.3614]	3
	**I267^{ECL2}L**	1.1 ± 0.0 [0.5812]	1.0 ± 0.0 [>0.9999]	0.9 ± 0.0 [0.9284]	0.8 ± 0.0 [>0.9999]	1.0 ± 0.0 [0.0841]	1.0 ± 0.1 [0.6959]	1.1 ± 0.0 [0.6278]	3
	**D266^{ECL2}E**	0.7 ± 0.0** [0.0042]	1.1 ± 0.1 [0.8927]	0.9 ± 0.1 [>0.9999]	0.8 ± 0.1 [>0.9999]	1.4 ± 0.1 [0.8747]	1.3 ± 0.1 [0.8924]	1.5 ± 0.2 [0.8847]	3
	**Quadruple^{TM1,7}**	0.6 ± 0.1** [0.0027]	1.2 ± 0.1 [0.2854]	1.1 ± 0.0 [0.1501]	0.9 ± 0.0 [0.4063]	1.8 ± 0.1* [0.0394]	1.6 ± 0.1 [0.1379]	1.8 ± 0.1* [0.0490]	3

Mutations are shown in the numbering of the cannabinoid CB_1 receptor (CB_1R) amino acid sequence as well as the Ballesteros and Weinstein GPCR numbering system. [a] Basal activity is expressed as fold over WT. Agonist-mediated activation values were obtained from [^{35}S]GTPγS assays on HEK293T membranes transiently expressing CB_1R constructs using 10 µM agonist, and are expressed as fold over its basal. Values are presented as the mean ± SEM of three independent experiments performed in duplicate (*n* indicates the number of biological replicates). One-way Welch ANOVA with Dunnett's T3 posthoc test was used to analyze differences in activation values compared to WT (*p < 0.05, ** p < 0.01, *** p < 0.001, **** p < 0.00001). Exact p values are given between square brackets. n.d. is not determined. Source data are provided as a Source Data file.
