# Supplementary material for: Structural basis of selective cannabinoid CB2 receptor activation
Source: Nat Commun. 2023 Mar 15;14:1447. doi: 10.1038/s41467-023-37112-9 (PMC10017709; doi:10.1038/s41467-023-37112-9)
Supplement: Supplementary file 2 — Reporting Summary [file 41467_2023_37112_MOESM2_ESM.pdf]

nature portfolio

Corresponding author(s): Tian Hua, Laura H. Heitman, Mario van der Stelt, Pal Pacher, Zhi-Jie Liu

Last updated by author(s): Feb 15, 2023

Reporting Summary

Nature Portfolio wishes to improve the reproducibility of the work that we publish. This form provides structure for consistency and transparency in reporting. For further information on Nature Portfolio policies, see our Editorial Policies and the Editorial Policy Checklist.

Statistics

For all statistical analyses, confirm that the following items are present in the figure legend, table legend, main text, or Methods section.

n/a	Confirmed	
☐	☒	The exact sample size (n) for each experimental group/condition, given as a discrete number and unit of measurement
☐	☒	A statement on whether measurements were taken from distinct samples or whether the same sample was measured repeatedly
☐	☒	The statistical test(s) used AND whether they are one- or two-sided<br>*Only common tests should be described solely by name; describe more complex techniques in the Methods section.*
☒	☐	A description of all covariates tested
☐	☒	A description of any assumptions or corrections, such as tests of normality and adjustment for multiple comparisons
☐	☒	A full description of the statistical parameters including central tendency (e.g. means) or other basic estimates (e.g. regression coefficient) AND variation (e.g. standard deviation) or associated estimates of uncertainty (e.g. confidence intervals)
☐	☒	For null hypothesis testing, the test statistic (e.g. F, t, r) with confidence intervals, effect sizes, degrees of freedom and P value noted<br>*Give P values as exact values whenever suitable.*
☒	☐	For Bayesian analysis, information on the choice of priors and Markov chain Monte Carlo settings
☒	☐	For hierarchical and complex designs, identification of the appropriate level for tests and full reporting of outcomes
☒	☐	Estimates of effect sizes (e.g. Cohen's d, Pearson's r), indicating how they were calculated

Our web collection on statistics for biologists contains articles on many of the points above.

Software and code

Policy information about availability of computer code

Data collection	Cryo-EM single particle data was automatically collected on the Titan Krios using serialEM 3.7.3.
Data analysis	CryoSPARC3.3.2, Phenix1.20.1-4487, COOT0.9.6, UCSF Chimera1.16, PyMOL 2.5.2, GraphPad Prism 6.0 and 9.0, ChemDraw 19.0

For manuscripts utilizing custom algorithms or software that are central to the research but not yet described in published literature, software must be made available to editors and reviewers. We strongly encourage code deposition in a community repository (e.g. GitHub). See the Nature Portfolio guidelines for submitting code & software for further information.

Data

Policy information about availability of data

All manuscripts must include a data availability statement. This statement should provide the following information, where applicable:
- Accession codes, unique identifiers, or web links for publicly available datasets
- A description of any restrictions on data availability
- For clinical datasets or third party data, please ensure that the statement adheres to our policy

The atomic coordinates for CB2-APD371-Gi-scFv16, CB2-CP55940-Gi-scFv16, CB2-HU308-Gi-scFv16 and CB2-LEI-102-Gi-scFv16 have been deposited in the Protein Data Bank with the accession codes 8GUQ, 8GUR, 8GUS and 8GUT. The EM maps for CB2-APD371-Gi-scFv16, CB2-CP55940-Gi-scFv16, CB2-HU308-Gi-scFv16 and CB2-LEI-102-Gi-scFv16 have been deposited in EMDB with the codes EMD-34276, EMD-34277, EMD-34278 and EMD-34279, respectively.

Human research participants

Policy information about studies involving human research participants and Sex and Gender in Research.

Reporting on sex and gender	N/A
Population characteristics	N/A
Recruitment	N/A
Ethics oversight	N/A

Note that full information on the approval of the study protocol must also be provided in the manuscript.

Field-specific reporting

Please select the one below that is the best fit for your research. If you are not sure, read the appropriate sections before making your selection.

☒ Life sciences ☐ Behavioural & social sciences ☐ Ecological, evolutionary & environmental sciences

For a reference copy of the document with all sections, see nature.com/documents/nr-reporting-summary-flat.pdf

Life sciences study design

All studies must disclose on these points even when the disclosure is negative.

Sample size	Sample sizes were determined by the current standard for in vitro binding and signaling experiments. Receptor expression experiments (ELISA) were carried out in two independent experiments and each datapoint was measured in quintuplicate. Binding and functional experiments were performed in at least three independent experiments performed in duplicate. These numbers are deemed large enough to identify variations between drug treatment or mutants as based on other studies with similar methodology. For cryo-EM experiment, the accurate micrographs and particles were included in the Method.
Data exclusions	No data were excluded.
Replication	Experiments were repeated as described above and in the manuscript sections methods or figure legends.
Randomization	Compounds were tested in dose-response curves on membrane fractions. All data was normalized to baseline/vehicle conditions within the plate and averaged among replicates. Compound or receptor effects were randomized on the plate to avoid 'plate effects'. Wild-type controls were always included on the plate in experiments with mutant receptors.
Blinding	There was no blinding in the study, the experiments for cryo-EM, in vitro and in vivo studies do not need blinding due to the purposes of the experiments.

Reporting for specific materials, systems and methods

We require information from authors about some types of materials, experimental systems and methods used in many studies. Here, indicate whether each material, system or method listed is relevant to your study. If you are not sure if a list item applies to your research, read the appropriate section before selecting a response.

Materials & experimental systems

n/a	Involved in the study	
☐	☒	Antibodies
☐	☒	Eukaryotic cell lines
☒	☐	Palaeontology and archaeology
☐	☒	Animals and other organisms
☒	☐	Clinical data
☒	☐	Dual use research of concern

Methods

n/a	Involved in the study	
☒	☐	ChIP-seq
☒	☐	Flow cytometry
☒	☐	MRI-based neuroimaging

Antibodies

Antibodies used	Monoclonal ANTI-FLAG® M2 antibody (Sigma Aldrich, #F3165), Peroxidase AffiniPure Goat Anti-Mouse IgG (H+L)(Jackson ImmunoResearch Laboratories, #115-035-003)
Validation	The former antibody has been validated for use in ELISA previously (https://www.sigmaaldrich.com/HK/zh/product/sigma/f3165); The later antibody has been validated for use in ELISA previously (https://www.jacksonimmuno.com/catalog/products/115-035-003)

Eukaryotic cell lines

Policy information about cell lines and Sex and Gender in Research

Cell line source(s)	Spodoptera frugiperda (Sf9) insect cells were used for CB2R-Gi co-expression for cryo-EM studies. Spodoptera frugiperda (Sf9) cells were a gift from Beili Wu lab from Shanghai Institute of Materia Medica, Chinese Academy of Sciences. Human embryonic kidney 293 T (HEK293T; female) cells for transfections were obtained from ATCC and CHO cells stably expressing hCB2R (CHOK1_hCB2bgal; PathHunter EA Parental Cell line, female) were obtained from DiscoverX.
Authentication	Cells were not further authenticated. Expression of wild-type or mutant CBR after transfection was authenticated by ELISA using the antibodies described above.
Mycoplasma contamination	All cells have been tested as negative for mycoplasma contamination.
Commonly misidentified lines (See ICLAC register)	No commonly misidentified cell lines were used.

Animals and other research organisms

Policy information about studies involving animals; ARRIVE guidelines recommended for reporting animal research, and Sex and Gender in Research

Laboratory animals	Ten to twelve-week-old male/female C57BL/6J mice from The Jackson Laboratory (Bar Harbor, ME, USA).
Wild animals	No wild animals were used in this study.
Reporting on sex	Both male and female mice were used according to NIH guidelines
Field-collected samples	no
Ethics oversight	All animal experiments reported in this manuscript complied with the National Institutes of Health "Guide for the Care and Use of Laboratory Animals" (NIH publication 86-23 revised 1985) and were approved by the Institutional Animal Care and Use Committee of the National Institute on Alcohol Abuse and Alcoholism (Bethesda, MD).Animals were kept in the institute's designated animal facility under constant temperature (22 ± 2 °C) and humidity, with 12-hour alternating light and dark cycles.

Note that full information on the approval of the study protocol must also be provided in the manuscript.
